# Hydration, water requirements, and energy balance from spring to summer in free-living older adults: a doubly labelled water study

**DOI:** 10.1038/s41598-026-38832-w

**Published:** 2026-02-19

**Authors:** Hyeon-Ki Kim, Yui Nakayama, Tsukasa Yoshida, Keiichi Yokoyama, Yuya Watanabe, Aya Itoi, Eiichi Yoshimura, Hinako Nanri, Rie Tsutsumi, Yumi Nakamura, Norifumi Tateishi, Rei Ono, Misaka Kimura, Hiroyuki Sagayama, Yosuke Yamada

**Affiliations:** 1 Medicine and Science in Sports and Exercise, Graduate School of Medicine, Tohoku University, Miyagi, Japan; 2 National Institutes of Biomedical Innovation, Health and Nutrition, Osaka, Japan; 3 Hiroshima University Graduate School of Biomedical and Health Sciences, Hiroshima, Japan; 4 Sports and Health Sciences, Graduate School of Biomedical Engineering, Tohoku University, 6-6-12, Aramaki Aza Aoba Aoba-ku, Myagi, 980-8579 Japan; 5 Co-creation Research Center for Water and Health Sciences, Suntory Global Innovation Center and Tohoku University, Miyagi, Japan; 6 Institute for Active Health, Kyoto University of Advanced Science, Kyoto, Japan; 7 Faculty of Sport Study, Biwako Seikei Sport College, Shiga, Japan; 8 Department of Health, Sports and Nutrition, Faculty of Health and Welfare, Kobe Women’s University, Hyogo, Japan; 9 Graduate School of Health Science, Kobe University, Hyogo, Japan; 10 Suntory Global Innovation Center Limited, Research Institute, Kyoto, Japan; 11 Institute of Health and Sports Sciences, University of Tsukuba, Ibaraki, Japan; 12 Specified Non-Profit Corporation Genki-Up AGE Project, Kyoto, Japan; 13 Corporation of Japan Dental Hygienists’ Association, Kyoto, Japan; 14 Kyoto Dietetic Association, Kyoto, Japan; 15 Faculty of Nursing, Doshisha Women’s College of Liberal Arts, Kyoto, Japan; 16 Faculty of Health and Sports Science, Doshisha University, Kyoto, Japan; 17 Department of Nutrition and Metabolism, National Institute of Health and Nutrition, National Institutes of Biomedical Innovation, Health and Nutrition, Osaka, Japan; 18 Laboratory of Applied Health Sciences, Kyoto Prefectural University of Medicine, Kyoto, Japan; 19 Graduate School of Environmental Studies, Tohoku University, Miyagi, Japan; 20 Faculty of Human Sciences, University of Tsukuba, Tokyo, Japan; 21 National Center for Geriatrics and Gerontology, Aichi, Japan; 22 Department of Dentistry and Oral-Maxillofacial Surgery, School of Medicine, Fujita Health University, Aichi, Japan; 23 Rehabilitation Clinic for Speech and Swallowing Disorders, The Nippon Dental University, Tokyo, Japan; 24 Faculty of Data Science, Shiga University, Shiga, Japan

**Keywords:** Physiology, Health care

## Abstract

The escalating effects of climate change, particularly global warming, are posing an increasing burden on human health. Older adults are particularly susceptible to the impact of extreme heat. Adequate water intake is essential to prevent dehydration in hot environments. Therefore, it is important to understand water turnover (WT) and intake. WT of older adults in hot environments remains unknown. This study aimed at investigating the seasonal effects on WT, total energy expenditure (TEE), and physical activity using doubly labeled water (DLW) and a triaxial accelerometer. A total of 26 older Japanese adult males and females aged ≥ 65 years participated in the study. WT and TEE were measured using DLW in May and August 2012. The mean values of maximum, mean, and minimum temperatures and mean humidity of the measurement days were 24 °C, 19 °C, 14 °C, and 57% in May (spring) and 35 °C, 29 °C, 25 °C, and 66% in August (summer) 2012, respectively. The mean (standard deviation, SD) age of the participants was 73.7 (5.4) years. Total body water increased significantly from 31.1 (4.6) to 31.9 (5.2) kg (+ 0.8 kg, *P* = 0.009) from May to August. TEE decreased significantly from 2271 (280) to 2123 (470) kcal/day (- 149 kcal/d, *P* = 0.036), while WT increased significantly from 2.939 (0.625) to 3.579 (0.943) L/day (+ 0.640 L/d, *P* < 0.001). WT increased by 640 mL/day during summer compared to that during spring, when the average temperature was 19 °C. Our findings indicate that WT increases during hot weather in older adults, reflecting seasonal adaptation.

**Supplementary Information:**

The online version contains supplementary material available at 10.1038/s41598-026-38832-w.

Climate change-induced global warming leads to more frequent and prolonged heat events globally^1^, which in turn affects human health both directly and indirectly by increasing health-related risks such as mortality and noncommunicable diseases (i.e., cardiovascular and infectious diseases)^2–5^. A recent study, using empirical data from 732 sites in 43 countries, attributed 37.0% (range, 20.5–76.3%) of warm-season heat-related deaths to anthropogenic climate change^6^. Therefore, mitigating and adapting to climate change is necessary.

Older adults are among the populations who are at an increased risk of heat-related health outcomes due to both physiological vulnerabilities, such as reduced sweat production, and social factors, including limited mobility or social isolation^7^. Over the past two decades, heat-related deaths in people aged ≥ 65 years have increased by 70%^5,8,9^. Exposure to extreme heat waves challenges the physiological adaptability of older adults, with a decreased ability to regulate body temperature, decrease in sweating during heat stress, and impaired ability to increase the skin blood flow due to heat^5,10,11^. Exposure to heat in older adults increases the hospitalization risk due to fluid and electrolyte disorders, renal failure, urinary tract infections, septicemia, and heat stroke^12^. Additionally, the proportion of excess heat-related deaths to total deaths that extreme temperatures can explain is higher for people aged ≥ 65 years than that for other age groups; deaths due to cardiovascular and respiratory issues are reported to be the highest^13^. Moreover, during the heat wave in Europe in 2022, heat-related mortality increased sharply with age^14^. Therefore, older adults may be more vulnerable to the effects of climate change in areas where temperatures increase on hot days.

Water intake is essential for homeostasis, especially in older populations. Older adults are less sensitive to feelings of depletion and more susceptible to dehydration than younger people^15,16^. Older adults have lower total body water, primarily due to reduced lean body mass, along with other age-related physiological changes^17–19^. In addition, renal function declines with age, leading to reduced urine-concentrating and urine-diluting capacity. This is primarily due to decreased renal sensitivity to antidiuretic hormone, rather than impaired hormone secretion^20–22^. Consequently, they are at a higher dehydration risk^23^. Dehydration refers to the process of losing body water, while hypohydration is the resulting state of reduced body water content^24^. Typical symptoms include thirst, dizziness, nausea, fatigue, difficulty concentrating, and headaches. Severe cases may result in seizures, impaired consciousness, hypotension, and organ failure, which can be life-threatening^25–27^. Therefore, adequate water intake is essential to maintaining the health of older adults during hot weather.

Despite the physiological importance of water, water intake among people who are living their everyday lives outside controlled research conditions is not well-documented or fully understood. Recently, factors that can predict adequate water intake in older adults have been identified, such as sex, age, weight, fat-free mass, total energy expenditure (TEE), PAL, human development index, humidity, and temperature^17,28^. However, information regarding water turnover (WT), including daily water intake (DWI) due to changes in ambient temperature in older adults remains unknown. Human body composition, including body fat percentage and skeletal muscle mass, as well as energy expenditure and intake, are influenced by external environmental factors such as changes in temperature and seasonality^29–31^. In particular, fat-free mass and body fat percentage affect the body water content^17^. Therefore, WT may vary in response to changes in ambient temperature. To the best of our knowledge, no previous study has characterized WT and DWI in older adults in relation to seasonal changes. Therefore, this study aimed to assess the impact of seasonal changes on hydration, energy intake and expenditure, physical activity, DWI, and WT by accurately measuring water intake, energy intake and expenditure, physical activity, and water loss in older adults in free-living conditions. Measurements were obtained during cool spring (May: 14–24 °C) and hot summer (August: 25–35 °C) seasons in Japan. We hypothesized that hot weather would increase WT and water intake and decrease physical activity in older adults compared with cool spring. Additionally, identifying these factors will reform the public health policy, including the need for developing guidelines for the hydration of older adults in hot environments.

## Results

**Fig. 1:**
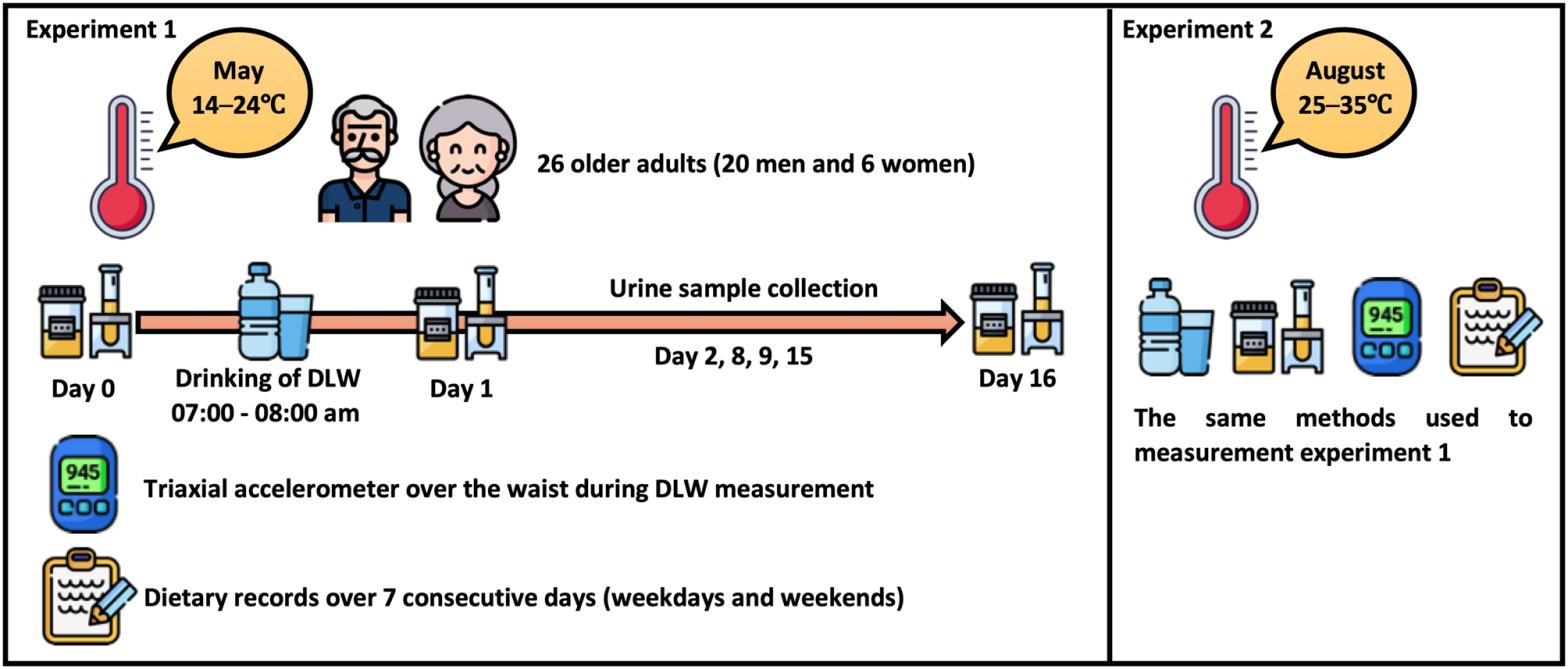
Experimental design of the study. DLW: doubly labeled water.

Figure 1 shows the experimental design of this study.

### Temperatures

The mean values of maximum, mean, and minimum temperature and mean humidity in the study area in which participants lived during the measurement period were calculated based on data published by the Japan Meteorological Agency. These were 24 °C, 19 °C, 14 °C, and 57%, respectively, in May (spring), and 35 °C, 29 °C, 25 °C, and 66%, respectively, in August (summer), in 2012^32^.

### Physical characteristics

The physical characteristics of the participants are presented in Table 1 (Supplementary Tables 1–3). Men and women did not significantly differ in weight and body mass index between the spring and summer (Supplementary Tables 2 and 3).

**Table 1:** Physical characteristics of the study participants.

Total (n = 26)	Spring (May)	Summer (Aug.)	Differences (Summer–Spring)	*P* value
Age [year]	73.7 ± 5.4			–
Height [cm]	160.7 ± 8.2			–
Weight [kg]	61.8 ± 7.8	62.2 ± 7.3	0.4 ± 1.2	0.081
BMI [kg/m^2^]	23.9 ± 2.3	24.1 ± 2.1	0.2 ± 0.5	0.067
DLW (n = 26)				
Total body water [kg]	31.1 ± 4.6	31.9 ± 5.2	0.8 ± 1.4	**0.009**
Water turnover [L/day]	2.939 ± 0.625	3.579 ± 0.943	0.640 ± 0.554	** < 0.001**
*TEEDLW [kcal/day]	2271.4 ± 280.2	2122.5 ± 470.0	–148.9 ± 342.6	**0.036**
*PALDLW [–]	1.**851** ± 0.195	1.726 ± 0.295	–0.125 ± 0.287	**0.040**
Physical activity (n = 25)				
Step counts [steps/day]	7288.6 ± 2710.7	6493.2 ± 2616.8	–795.4 ± 1628.8	**0.022**
LPA [min/day]	366.2 ± 103.5	323.5 ± 73.4	–42.7 ± 49.7	** < 0.001**
MVPA [min/day]	43.0 ± 23.6	38.4 ± 24.1	–4.6 ± 19.1	0.243
MVPA [met･hr/wk]	14.9 ± 10.2	14.1 ± 11.5	–0.8 ± 8.8	0.662
SBSD [min/day]	1030.8 ± 109.0	1078.1 ± 80.5	47.3 ± 57.2	** < 0.001**
#TEEACC [kcal/day]	2116.8 ± 225.6	2042.1 ± 215.8	–74.7 ± 116.3	**0.004**
#AEEACC [kcal/day]	664.2 ± 147.7	597.0 ± 128.5	–67.2 ± 104.7	**0.004**
#PALACC [–]	1.712 ± 0.147	1.650 ± 0.121	–0.062 ± 0.094	**0.003**
Dietary intake (n = 22)				
Energy intake [kcal/day]	1924.7 ± 286.3	1788.0 ± 304.3	–136.7 ± 161.1	**0.001**
Protein [g/day]	77.7 ± 13.9	71.4 ± 16.3	–6.3 ± 9.3	**0.005**
Fat [g/day]	56.5 ± 14.7	50.2 ± 16.0	–6.3 ± 10.0	**0.008**
Carbohydrate [g/day]	273.1 ± 36.3	258.3 ± 44.5	–14.9 ± 32.8	**0.045**
Sodium [g/day]	10.1 ± 2.2	10.0 ± 1.8	–0.1 ± 1.7	0.852
Daily water intake (n = 22)				
Metabolic water [L/day]	0.30 ± 0.03	0.28 ± 0.07	–0.02 ± 0.05	0.104
Inspiratory water [L/day]	0.071 ± 0.008	0.128 ± 0.029	0.057 ± 0.024	** < 0.001**
Transcutaneous water [L/day]	0.10 ± 0.01	0.19 ± 0.02	0.09 ± 0.01	** < 0.001**
Water in ingested foods [L/day]	0.97 ± 0.22	0.95 ± 0.29	–0.02 ± 0.24	0.703
Water in ingested fluids [L/day]	1.58 ± 0.69	2.14 ± 0.95	0.55 ± 0.47	** < 0.001**
Water fluids/foods + fluids ratio [%]	59.48 ± 14.55	67.13 ± 12.46	7.51 ± 9.68	**0.002**

All data are presented as mean ± standard deviation.

*P*-values in bold indicate significant differences in the variables between spring and summer, as calculated using the paired Student’s *t* test. BMI, body mass index; DLW, doubly labeled water; ACC, triaxial accelerometer; TEE, total energy expenditure; PAL, physical activity level; AEE, physical activity energy expenditure; LPA, light physical activity; MVPA, moderate-to-vigorous physical activity; SBSD, sedentary behavior including sleep duration. *Asterisk: TEE_DLW_ and PAL_DLW_ calculated using DLW; ^#^Hashtag: TEE_ACC_, AEE_ACC_, and PAL_ACC_ calculated using ACC.

### Comparison of TBW level and WT between spring and summer

**Fig. 2:**
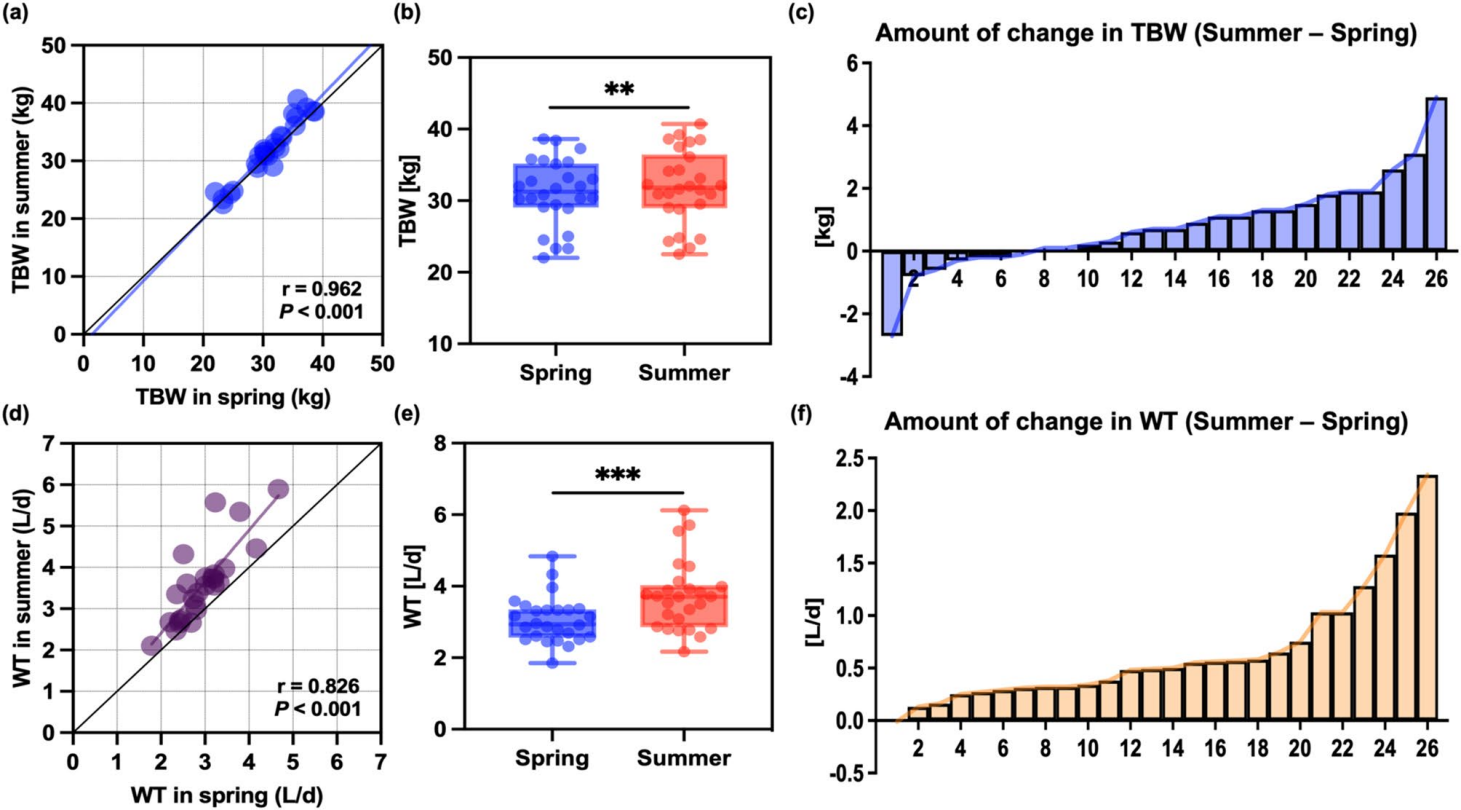
Association between TBW and WT in spring and summer (n = 26). Association between spring WT and TBW and summer WT and TBW (**a** and **d**), Comparison of TBW and WT between the spring and summer experiments (**b** and **e**), **P < 0.01, ***P < 0.001 compared to TBW and WT in the summer experiment (paired Student’s *t*-test), amount of change in TBW and WT for each participant (c and f). TBW, total body water; WT, water turnover.

Significantly higher total body water (TBW) levels (spring: 31.1 ± 4.6 kg vs. summer: 31.9 ± 5.2 kg; differences: 0.8 ± 1.4 kg,* P* = 0.009)　and WT　(spring 2.939 ± 0.625 L/day vs summer 3.579 ± 0.943 L/day, differences 0.640 ± 0.554 L/day,* P* < 0.001) were observed in summer than those in spring (Fig. 2b,e, Table 1, Supplementary Tables 4–6). Furthermore, the TBW level increased in summer compared with that in spring in 19 out of the 26 participants (Fig. 2c, Supplementary Table 4). In contrast, WT was confirmed to increase in summer for all the participants except for one compared with that in spring (Fig. 2f, Supplementary Table 4). In addition, TBW levels (r = 0.962, *P* < 0.001) and WT (r = 0.824, *P* < 0.001) showed strong correlations between spring and summer, respectively (Fig. 2a,d).

### Comparison of DWI between spring and summer

Inspiratory and transcutaneous water intakes in older adults were significantly higher in summer than in spring (Table 1; both* P* < 0.001). Water intake from food showed no significant difference between spring and summer; however, water intake from fluids (spring 1.58 ± 0.69 L/day vs summer 2.14 ± 0.95 L/day, differences 0.55 ± 0.47 L/day)　was significantly higher in summer than in spring (Table 1; *P* < 0.001). Furthermore, the proportion of water intake from fluids was significantly higher in summer compared with that in spring (approximately 70% vs approximately 60%, respectively; Table 1; *P* = 0.002; Supplementary Tables 7–9).

### Comparison of PAL between spring and summer

**Fig. 3:**
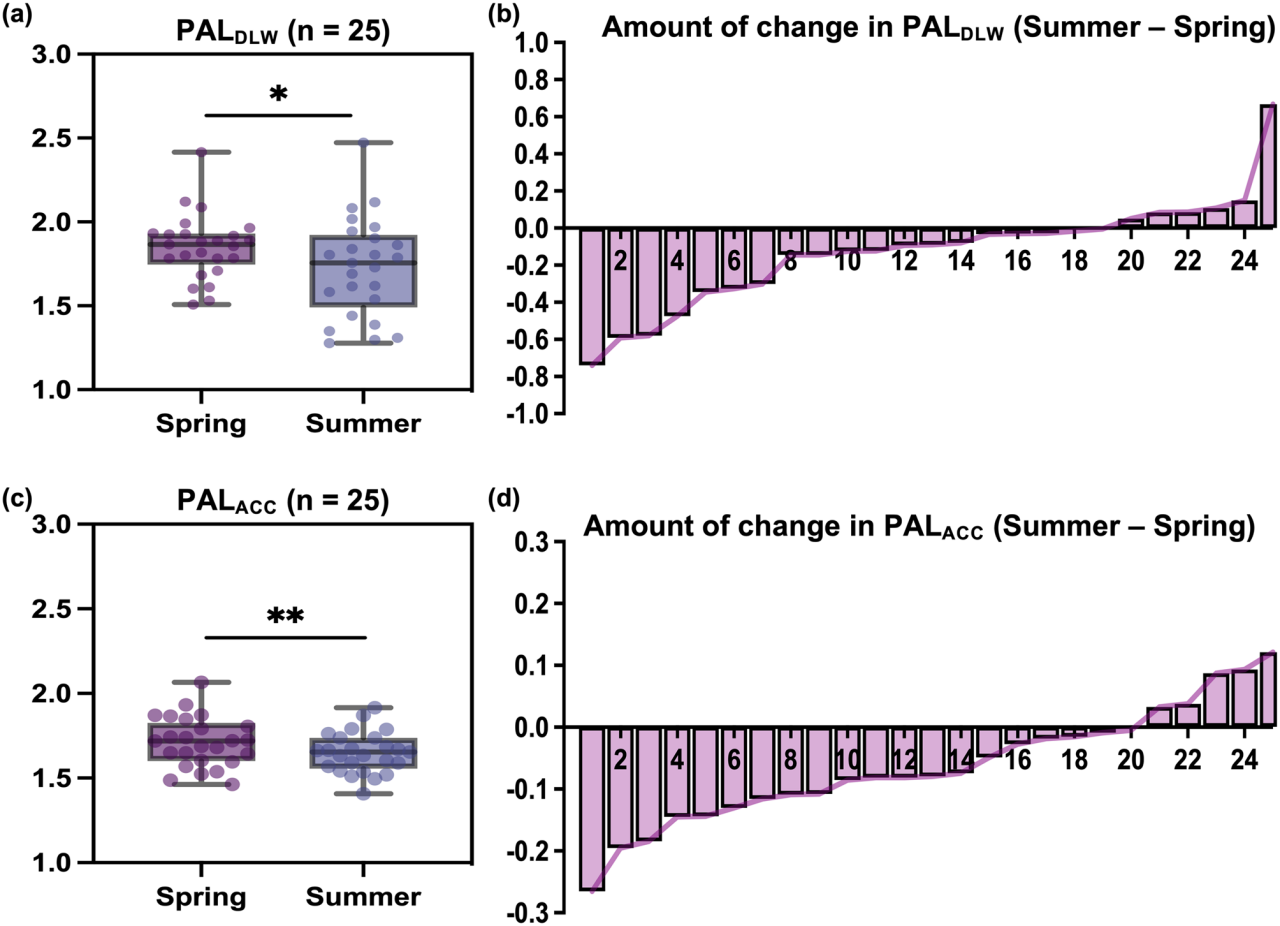
Comparison of PAL in spring and summer (n = 25). Comparison of PAL between the spring and summer experiments calculated by DLW (**a**), comparison of PAL between the spring and summer experiments calculated by ACC (**c**), *P < 0.05, **P < 0.01, compared to PAL in the summer trial (paired Student’s *t*-test), and amount of change in PAL calculated by DLW or ACC for each participant (**b** and **d**). ACC, triaxial accelerometer; DLW, doubly labeled water; PAL, physical activity level.

**Fig. 4:**
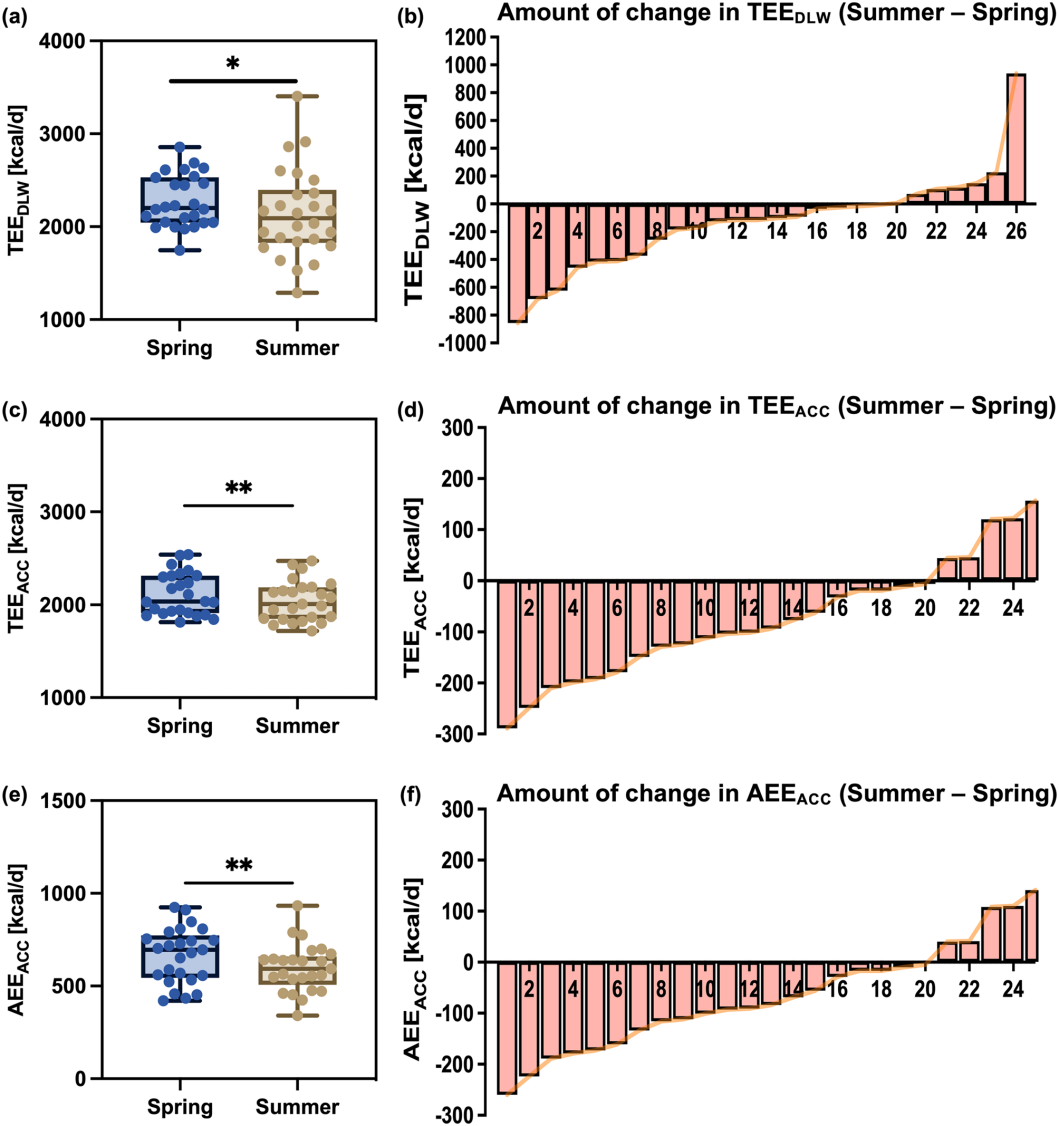
Comparison of TEE and AEE between spring and summer. Comparison of spring and summer TEE experiments calculated by DLW (n = 26), comparison of spring and summer experiments of TEE and AEE calculated by ACC (n = 25), *P < 0.05, **P < 0.01, compared to TEE and AEE in the summer experiment (paired Student’s *t*-test), the amount of change in TEE and AEE calculated by DLW or ACC for each participant (**b**, **d**, and **f**); TEE: total energy expenditure; ACC: triaxial accelerometer; AEE: physical activity energy expenditure; DLW: doubly labeled water.

Step count (spring 7288.6 ± 2710.7 steps/day vs summer 6493.2 ± 2616.8 steps/day; differences −795.4 ± 1628.8 steps/day,* P* = 0.022)　and light-physical activity (LPA) (spring 366.2 ± 103.5 min/day vs summer 323.5 ± 73.4 min/day; differences −42.7 ± 49.7 min/day;* P* < 0.001)　were significantly lower in summer than those in spring (Table 1, Supplementary Tables 10–12). However, moderate-to-vigorous physical activity (MVPA) showed no significant differences between the two experiments. In contrast, sedentary behavior, including sleep duration (SBSD) was significantly higher in summer than in spring　(spring 1030.8 ± 109.0 min/day vs summer 1078.1 ± 80.5 min/day; differences 47.3 ± 57.2 min/day;* P* < 0.001; Table 1). Examining PAL using DLW　(spring 1.851 ± 0.195 vs summer 1.726 ± 0.295; differences: 0.125 ± 0.287;* P* < 0.05) and a triaxial accelerometer (spring 1.712 ± 0.147 vs. summer 1.650 ± 0.121, differences = 0.062 ± 0.094,* P* < 0.01)　revealed a significant decrease in summer compared with that in spring (Fig. 3a,c, Table 1). Examination of PAL change for each participant confirmed that PAL decreased for most participants in summer compared to that in spring (Fig. 3b,d). TEE was confirmed using DLW and a triaxial accelerometer. TEE calculated using DLW showed a significant decrease in summer compared with that in spring (spring 2271.4 ± 280.2 kcal/day vs summer 2122.5 ± 470.0 kcal/day; differences −148.9 ± 342.6 kcal/day;* P* = 0.036; Fig. 4a, Table 1). Furthermore, TEE and physical activity energy expenditure (AEE) calculated using triaxial accelerometers showed significant decreases (TEE, spring 2116.8 ± 225.6 kcal/day vs summer 2042.1 ± 215.8 kcal/day; differences −74.7 ± 116.3 kcal/day;* P* < 0.01; and AEE, spring 664.2 ± 147.7 kcal/day vs summer 597.0 ± 128.5 kcal/day; differences −67.2 ± 104.7 L/day;* P* < 0.01)　in summer compared with those in spring (Fig. 4c,e, Table 1). Analysis of the amount of change for each participant also revealed that the TEE and AEE decreased for many participants in summer compared to those in spring (Fig. 4b,d,f).

### Comparison of energy intake between spring and summer

We next examined energy intake in spring and summer using 7-day food records, including weekdays and holidays. The daily total energy　(spring 1924.7 ± 286.3 kcal/day vs summer 1788.0 ± 304.3 kcal/day; differences −136.7 ± 161.1 kcal/day;* P* = 0.001), protein　(spring 77.7 ± 13.9 g/day vs. summer 71.4 ± 16.3 g/day; differences −6.3 ± 9.3 g/day;* P* = 0.005), fat　(spring 56.5 ± 14.7 g/day vs. summer 50.2 ± 16.0 g/day; differences −6.3 ± 10.0 g/day; *P* = 0.008), and carbohydrate (spring 273.1 ± 36.3 g/day vs summer 258.3 ± 44.5 g/day; differences −14.9 ± 32.8 g/day;* P* = 0.045)　intake were significantly reduced in summer compared with those in spring (Table 1, Supplementary Tables 13–15). However, sodium intake did not show a significant difference between summer and spring.

### Association between the amount of change in PAL, TEE, and WT

**Fig. 5:**
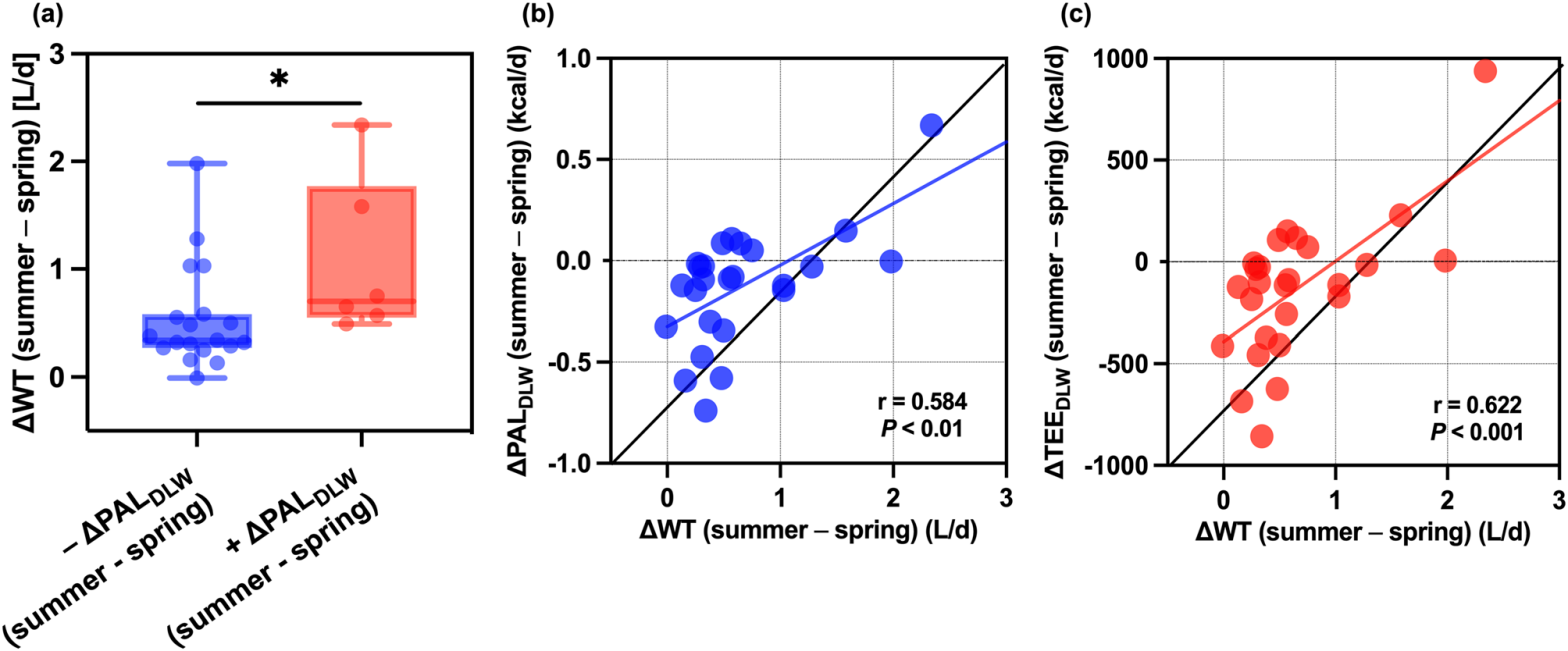
Relationship between the amount of change in TEE and PAL calculated using DLW and the amount of change in WT (n = 25). Comparison of the amount of change in WT due to different PAL variations in the spring and summer experiments. (**a**) Negative delta PAL_DLW_ indicates participants who experienced a decrease in PAL in summer compared with that in spring (n = 19). In contrast, a positive delta PAL_DLW_ indicates that participants experienced an increase in PAL in summer compared to spring (n = 6), *P < 0.05, compared to the amount of change in WT in the summer experiment (Mann–Whitney). Partial correlation coefficients were calculated between the amount of change in WT and the amount of change in PAL and TEE, with sex as the adjustment variable (**b** and **c**). Delta values represent the difference between summer and spring. DWL, doubly labeled water; PAL, physical activity level; TEE, total energy expenditure; WT, water turnover.

Next, while elucidating the relationship between water intake and energy expenditure in hot environments, we observed a significant positive association between the amount of change in WT and those in PAL_DLW_ (r = 0.584; *P* < 0.01) and TEE_DLW_ (r = 0.622; *P* < 0.001; Fig. 5b,c). In addition, participants whose summer PAL_DLW_ increased showed a significantly higher change in WT than those whose summer PAL_DLW_ decreased (Fig. 5a; *P* < 0.05).

## Discussion

In this study, we longitudinally compared WT, TBW, energy expenditure/intake, and physical activity in older adults in spring (maximum temperature, 24 °C) and summer (35 °C) using DLW. Energy expenditure, DWI, WT, and TBW levels increased in summer compared with those in spring, whereas physical activity and energy intake decreased. Furthermore, participants whose PAL_DLW_ increased in summer compared to those in spring showed a greater change in their WT than those whose PAL_DLW_ decreased. Our findings suggested that the WT of older adults increases during hot weather, reflecting seasonal differences.

Previous studies have reported seasonal changes in physical activity and water intake^33–36^. However, only a few longitudinal studies have been conducted on seasonal changes in WT and TBW levels under free-living conditions. Water requirements are influenced by many factors, including physiological factors, such as race, age, sex, and physical activity, and environmental factors, such as season and climate^36–41^. Recently, growing concerns regarding health risks are evident due to the effects of global warming and climate change^1,2^. Heat-related illnesses and mortality rates have increased owing to the impact of climate change^2–5^. In particular, as humans age, the sense of thirst, vasodilation, and sweat gland function decreases; therefore, adequate water intake is essential for maintaining health in older adults who are highly susceptible to heat stress^18,37^. The high water requirements of humans can lead to dehydration owing to many contributing factors, such as climate, physical activity, and diet. This study demonstrated that WT and TBW in older adults increased in summer compared to those in spring, indicating that these variables exhibit seasonal variation. These findings reflect physiological responses to seasonal changes in older adults, suggesting that temperature fluctuations may influence hydration needs in this population.

In older adults, PAL and TEE were lower in summer (hot environment) than in spring. A previous study of young adults examined WT, PAL, and energy intake during summer and winter. In men, water loss did not differ between summer and winter, but in women, water loss was greater during summer than that during winter^34^. Additionally, energy intake was lower and physical activity was higher in summer than in winter^34^. In our study, WT was higher in summer than in spring for both men and women (Supplementary Tables 5 and 6); our findings contradict with those of a previous study^34^. Energy intake was higher in spring than in summer, but physical activity was lower in summer than in spring. This discrepancy between the findings of the two studies can be attributed to the difference in the ambient temperature range. In another study conducted in the Netherlands, a comparison was carried out between summer and winter; however, given the characteristics of the region based on the Köppen climate classification, it is possible that extreme temperatures were not considered^42^. In our study, the maximum daily temperature in summer was 35 °C. Public health recommendations advise people who are more susceptible to heat, such as older adults, to avoid going outdoors or engaging in strenuous physical activity during the hottest times of the day^43^, which may explain some of the physical activity findings.

When examining the relationship between physical activity and fluid intake, it is important to consider the seasonality of physical activity. In particular, decreased physical activity in older adults is associated with frailty and risk of developing chronic diseases. Therefore, it is crucial for older adults to maintain and increase physical activity^44,45^. This reduction in TEE and increase in WT observed during summer could be interpreted as a short-term adaptive response in older adults to mitigate heat stress, as public health recommendations often advise older individuals to limit outdoor activities and increase fluid intake during hot weather. While such behavioral adjustments may be beneficial in the short term, prolonged reductions in physical activity levels during increasingly frequent and intense heat events could have detrimental long-term consequences, including accelerated functional decline and increased risk of chronic diseases. As global temperatures continue to rise, these seasonal behavioral changes might become more pronounced, potentially leading to further reductions in physical activity among older adults. This highlights the need for public health strategies that balance protection from heat with the maintenance of adequate physical activity to preserve health and functional capacity in aging populations^46^.

In this study, the participants whose PAL_DLW_ increased in summer showed a greater change in WT than those whose PAL_DLW_ decreased. Accordingly, participants who exhibited increased physical activity also showed greater changes in WT, suggesting a potential association between physical activity and WT. On the other hand, excessive water intake may pose health risks such as hyponatremia, particularly in older adults with impaired kidney function or those taking certain medications^47–49^. Furthermore, previous studies have suggested that older women may be more prone to excessive water consumption and may have a higher risk of developing hyponatremia compared to men^48,49^. Therefore, individualized hydration strategies should be considered. However, this study did not assess urinary sodium concentration or osmolality, which are important biochemical indicators of hydration status, including both dehydration and overhydration. This limitation should be taken into account when interpreting the findings related to hydration.

The decrease in energy intake during the summer in this study was consistent with the findings of previous studies. Previous meta-analyses and reviews have reported that energy intake is higher in winter than in the other seasons^31,50^. Ambient temperature, which varies greatly with seasonal change, affects the secretion of appetite-related hormones^51,52^. Cold temperatures increase the secretion of ghrelin, which stimulates appetite, and decrease the secretion of leptin, which suppresses appetite. In contrast, hot temperatures increase the levels of peptide YY, which suppresses appetite^51,52^. Only a few studies have examined the variations in appetite-related hormones according to seasons; however, blood leptin levels were reported to increase in summer compared to those in spring^53^. Therefore, seasonal temperature changes are believed to affect energy intake through inducing changes in the secretion of hormones and neurotransmitters that regulate appetite.

This study had some limitations. First, we focused only on community-dwelling, relatively healthy older adults, and did not include children or adults with medical conditions or mobility impairments. Therefore, generalizing this study findings to a larger population is not easy. Second, we did not include information on artificial temperature regulation. In Japan, air systems are installed at homes, offices, and public transportation facilities. A previous study reported no significant association between ambient temperature fluctuations and PAL, TEE, or AEE^38^. This could be attributed to the fact that the environment in which we live is adjusted to 18–25 °C, which is thought to maintain energy expenditure at constant levels. Third, dietary habits might have a greater effect on WT than on activity or environment. East Asians, such as the populations in Japan and Korea, might consume a diet that is high in water content^33,54^. In Japan, water intake from meals is higher than that in the United States and European countries^55–57^. Therefore, the effect of seasonal variation on WT may differ in areas with different cultural practices. Finally, the effects of PAL variation on physical fitness were not confirmed. A previous study revealed that seasonal variation affected PAL and AEE, but no effect was observed on physical fitness^35^. However, that study was conducted on participants in their 20 s and 30 s. As this study was conducted on adults aged ≥ 65 years, the effect of seasonal changes on physical fitness might be greater on this study cohort than in young adults. The current study unveiled that physical activity and energy expenditure decreased in older adults during hot summer compared with cool spring. A previous study showed that physical activity is associated with physical fitness in older adults^58^. In the future, it will be essential to examine the association between physical activity and fitness in older adults during seasonal variations to maintain and promote the health of the older population. Therefore, the association between seasonal changes in physical activity and fitness requires further investigation.

In conclusion, WT, TBW level, and DWI in older adults showed significant seasonal variations, where they increased in summer compared with those in spring. Water plays an essential physiological role in humans^18^. Therefore, it is necessary to consider the effects of WT seasonality on the association between water intake and health in older adults in future studies.

## Online methods

### Study participants

This study participants included 26 older adults (age 66–83 years; men, n = 20; women, n = 6) who lived in Kameoka City, Kyoto Prefecture, Japan (Fig. S1). This study used 7-day dietary records (DRs) and a triaxial accelerometer to assess energy intake and PAL. The exclusion criteria for the analysis of energy intake and PAL were as follows: (1) missing food records (n = 4) and (2) malfunctioning accelerometers (n = 1). Consequently, 22 and 25 participants were included in the analyses of energy intake and PAL, respectively.

This study was approved by the Ethics Committee of Kyoto Prefectural University of Medicine (approval no. RBMR-E-363) and the National Institute of Health and Nutrition (NIHN187-3). This study was conducted in accordance with the Declaration of Helsinki. Informed consent was obtained from all the participants after explaining the experimental details.

### Study design

This study consisted of two experiments to determine the differences between the spring and summer WT and TEE in older adults: the spring experiment was conducted in May, and the summer experiment was conducted in August. Japan Meteorological Agency defines spring and summer as the three months between March and May and between June and August, respectively^59^. The study period for each trial was approximately two weeks. In both experiments, the WT, TBW level, and TEE were measured using DLW. Physical activity was assessed using a triaxial accelerometer, and energy intake was calculated using the 7-day DRs (Fig. 1). The participants were asked to continue their normal lives without changing their lifestyle habits, such as diet and exercise.

Meteorological data were obtained from the Japan Meteorological Agency (JMA) website, based on official records from the Kyoto Local Meteorological Observatory (Station ID: 47,759)^32^, located approximately 15 km from the participants’ residential area (Kameoka City, Kyoto Prefecture). The temperature data represent standard dry-bulb air temperatures measured in the shade within a Stevenson screen at a height of 1.5 m, following World Meteorological Organization protocols. For the measurement periods in May (spring) and August (summer) 2012, daily maximum, mean, and minimum air temperatures, as well as mean relative humidity, were extracted.

### Measurement of anthropometry

Height and weight of the participants were measured to the nearest 0.1 cm and 0.1 kg, respectively, using a digital weight scale and stadiometer (DST-210S, Muratec-KDS Corp.; Kyoto, Japan). Body mass index (BMI) was calculated as weight in kilograms divided by the square of height in meters.

### Measurement of WT, TBW level, TEE, and PAL

WT, TBW level, and TEE were measured over 14 days using DLW. The details of these methods have been previously described^60,61^. Briefly, the participants arrived at the study facility on day 0, and a urine sample was acquired to measure baseline ^2^H and ^18^O accumulation. Between 07:00 and 08:00 am, a premixed dose containing approximately 0.12 g/kg estimated TBW of ^2^H_2_O (99.8 atom%; Taiyo Nippon Sanso; Tokyo, Japan) and 2.5 g/kg estimated TBW of H_2_^18^O (10.0 atom%; Taiyo Nippon Sanso; Tokyo, Japan) was administered to each participant. Urine samples were collected approximately 24 h after drinking (day 1) and in the morning of day 16. Aliquots of urine samples were stored at −15 ℃ for subsequent analysis using isotope ratio mass spectrometry (Hydra 20–20, SerCon Ltd.; Crewe, UK).

CO_2_ and H_2_ gases were used to equilibrate the ^18^O and ^2^H, respectively. A Pt catalyst was used to equilibrate ^2^H. The dilution spaces of ^18^O (No) and ^2^H (Nd) and elimination rates of ^18^O (ko) and ^2^H (kd) were obtained. The TBW (mol) was calculated as the mean of Nd (mol) divided by 1.043 and no (mol) divided by 1.007, which was corrected for isotopic sequestration in non-aqueous tissues. Carbon dioxide production rates (rCO_2_, mol/day) were calculated using the following equation: rCO_2_ = 0.4554 × TBW × (1.007 ko − 1.043 kd), where ko and kd represent the ^18^O and ^2^H elimination rates per day, respectively. The daily WT rate was calculated using the following equation: WT (L/day) = kd × Nd. TEE was determined using a modified Wier equation based on rCO_2_ (mol/day) and estimated 24-h respiratory quotients (RQ): TEE (kcal/day) = 22.6 × (3.9 × (rCO_2_/RQ) + 1.1 × rCO_2_), where 22.6 (L/mol) is the molar volume of CO_2_^62^. PAL was calculated as TEE/predicted basal metabolic rate (pBMR)^60^. The pBMR was estimated according to the fifth recommended dietary allowance for the Japanese people^63^.

### Estimation of DWI

DWI accounts for metabolic water, inspiratory water (moisture content of air-breathing), transcutaneous water intake (water absorbed through the skin), water intake from fluids (drinking water and other beverages), and food. These variables were calculated using the formulas described in a previous study^64^. In addition, the water content in the ingested fluids was calculated by subtracting the values of the following variables and water content in the ingested foods calculated by DR from the WT value. Moreover, water intake from fluids was calculated by subtracting the values of the following variables, and water intake from foods was calculated from the DRs of the WT value.

$$\begin{aligned} {\text{Metabolic }}\;{\text{water }}\left( {{\mathrm{L}}/{\mathrm{day}}} \right) = & {\text{TEE }}\left( {{\mathrm{kcal}}/{\mathrm{day}}} \right) \times ({1}/{1}0^{{5}} ) \\ & \times [\left( {\% \, \;{\mathrm{fat}} \times 0.{119}} \right) + \left( {\% \;{\text{ protein}} \times 0.{1}0{3}} \right) \\ & + \left( {\% \, \;{\mathrm{carbohydrates}} \times 0.{15}} \right) + \left( {\% \;{\text{ alcohol}} \times 0.{168}} \right)]. \\ \end{aligned}$$

%fat, %protein, and %carbohydrate were calculated based on values obtained from the DRs. Alcohol intake was zero, as alcohol consumption was prohibited throughout the study.

$$\begin{aligned} {\text{Inspiratory }}\;{\mathrm{water}}\;{\text{ intake }}\left( {{\mathrm{L}}/{\mathrm{day}}} \right) = & {\mathrm{respiratory}}\;{\text{ volume }}\left( {{\mathrm{L}}/{\mathrm{day}}} \right) \\ & \times {\mathrm{absolute}}\;{\text{ humidity}}/{1},000. \\ \end{aligned}$$

The average relative humidity values in the spring and summer experiments were 57% and 66%, respectively. The respiratory volume was calculated from the rCO_2_ obtained from DLW, assuming that 3.5% of the inhaled air was CO_2_.

$$\begin{aligned} {\mathrm{Transcutaneous}}\;{\text{ water }}\;{\text{intake }}\left( {{\mathrm{L}}/{\mathrm{day}}} \right) = & 0.{18} \\ & \times \left( {{\mathrm{absolute}}\;{\text{ humidity}}/{21}.{7}} \right) \\ & \times {\mathrm{body}}\;{\text{ surface}}\;{\text{ area }}\left( {{\mathrm{BSA}};{\text{ m}}^{{2}} } \right) \times {1}.{44}. \\ \end{aligned}$$

The BSA was calculated using the Dubois formula^65^. The clothing factor was set to 50% because clothing reduces the evaporation rate through the skin^64^.

### Measurement of physical activity

All participants were asked to wear a triaxial accelerometer (Actimarker, Panasonic, Osaka, Japan) over the waist for the entire 2-week period of each experiment, and the data were adjusted for the spring and summer DLW periods. The participants wore the triaxial accelerometer daily from morning to night, except during bathing and bedtime. Data were included from participants who wore the accelerometer for at least 10 h (600 min) per day. The assessments included MVPA, LPA, and the number of required steps. MVPA was defined as an activity with an intensity of ≥ 3 metabolic equivalents (METs) in a minute. In contrast, LPA was defined as an activity with an intensity equivalent to 1.6–2.9 METs in a minute. The 24-h average MET was obtained from the triaxial accelerometer, and PAL was calculated using the following equation: PAL = (24-h average METs × 1.1)/0.9^60^. TEE was calculated using the following equation, considering diet-induced thermogenesis as 10% of TEE: TEE (kcal/d) = (pBMR × 24-h average METs)/0.9^60^. Physical AEE was calculated as follows: AEE (kcal/day) = 0.9 × TEE – pBMR^60^.

### Dietary assessment

DR data were collected for seven consecutive days in May (spring) and August (summer) of 2012, including weekdays and weekends. In the preparation sessions, our team of research dietitians received instructions on how to guide the participants with respect to completing the DR, using previously completed dietary record sheets for reference. Each participant was provided with blank record sheets, a digital scale (TANITA, Tokyo, Japan), and paper instructions.

The research dietitians instructed the participants to carefully record all foods and beverages consumed daily, whether during or between meals. In cases where quantification was challenging (such as meals prepared in restaurants or purchased in supermarkets or convenience stores), participants were advised to make the most accurate measurements possible using household utensils.

To ensure data accuracy and reliability, the completed records were carefully reviewed by dietitians at the homes of participants and underwent at least two rounds of standardized checks. The collected DRs were then coded and recorded by the research dietitians into the WELLNESS21 software (TopBusinessSystem; Okayama, Japan), a specialized tool for energy and nutrient analysis. This software was aligned with the *standard tables of food composition in Japan*^66^. In cases where the foods or beverages reported by participants were not found in *the standard tables of food composition in Japan*, appropriate substitutions from similar food categories were used.

### Statistical analyses

Normal or non-normal distributions of the data were analyzed using the Shapiro–Wilk test. Paired Student’s *t* test was used to compare the physical characteristics of men and women. TBW, WT, DWI, physical activity (steps, LPA, MVPA, and SBST), PAL, and TEE were compared between the two trials. Conversely, for measures in which normality was not assumed, the Wilcoxon signed-rank test was used to compare variables between the two experiments. Mann–Whitney U test was used to compare the amount of change in WT between the participants whose summer PAL_DLW_ showed an increase and those whose PAL_DLW_ showed a decrease. Pearson’s product-moment correlation coefficients were calculated between spring WT and TBW and summer WT and TBW. Partial correlation coefficients were calculated between the amount of change in WT and the amount of change in PAL_DLW_ and TEE_DLW_, with sex as the adjustment variable. Post-hoc testing using G*Power (version 3.1.9.2; University of Kiel, Germany) showed a medium effect size (Cohen’s d = 0.6) for a sample size of 26 participants, with a power of 0.80 and a significance level of α = 0.05. All statistical procedures were performed using PASW Statistics 28.0 (IBM Corp., Armonk, NY, USA), with a significance level of < 5%.

## Supplementary Information

Supplementary Information 1.

Supplementary Information 2.

## Data Availability

The original contributions of this study are included in this article. Further inquiries can be directed to the corresponding author, Yosuke Yamada.
