# Supplementary figures and images for: Hydration, water requirements, and energy balance from spring to summer in free-living older adults: a doubly labelled water study
Source: Sci Rep. 2026 Feb 19;16:9872. doi: 10.1038/s41598-026-38832-w (PMC13018177; doi:10.1038/s41598-026-38832-w)

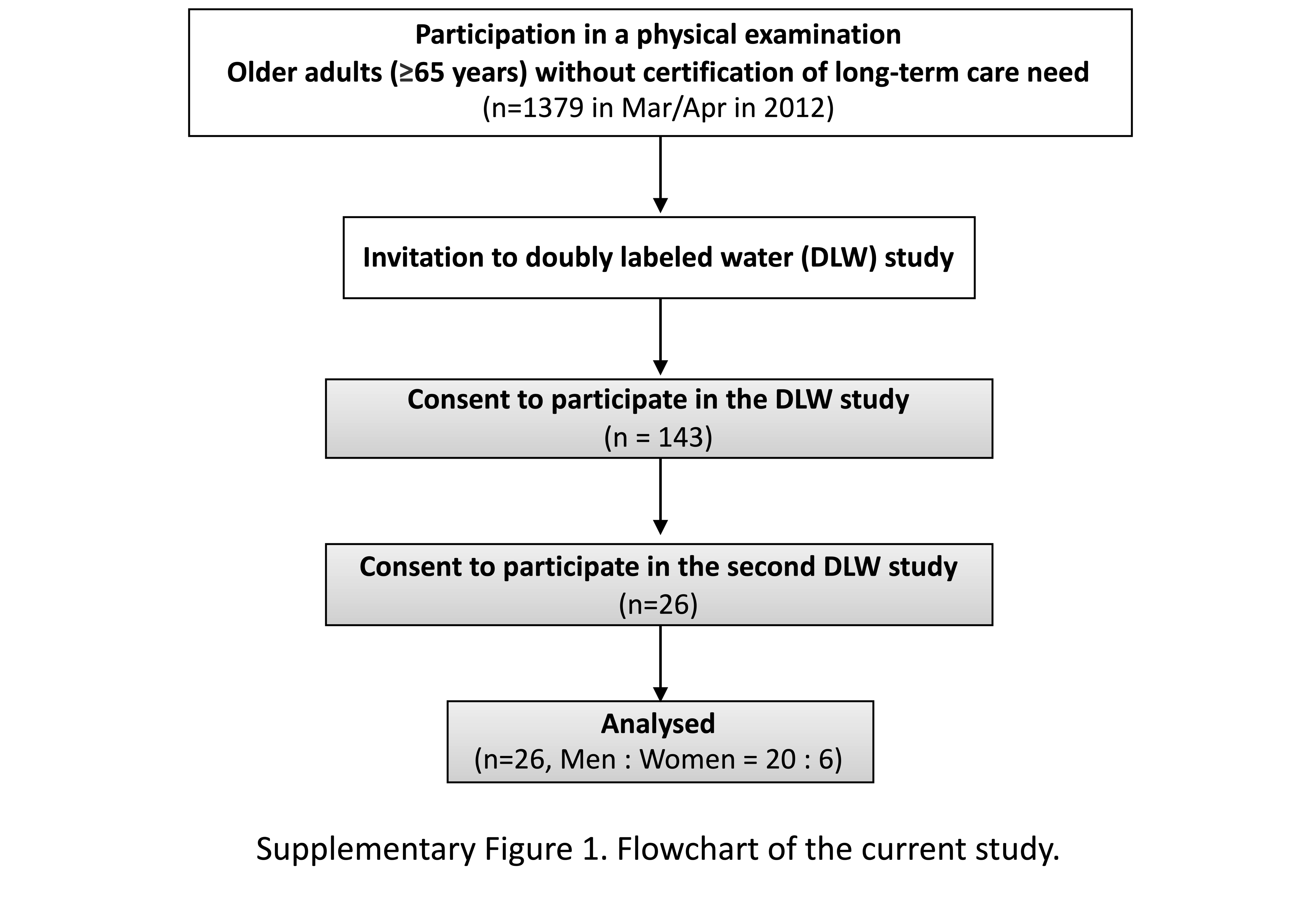

Supplement: Supplementary file 1 — Supplementary Information 1. [file 41598_2026_38832_MOESM1_ESM.tiff]
