# Supplementary material for: Hydration, water requirements, and energy balance from spring to summer in free-living older adults: a doubly labelled water study
Source: Sci Rep. 2026 Feb 19;16:9872. doi: 10.1038/s41598-026-38832-w (PMC13018177; doi:10.1038/s41598-026-38832-w)
Supplement: Supplementary file 2 — Supplementary Information 2. [file 41598_2026_38832_MOESM2_ESM.pptx]

## Slide 1
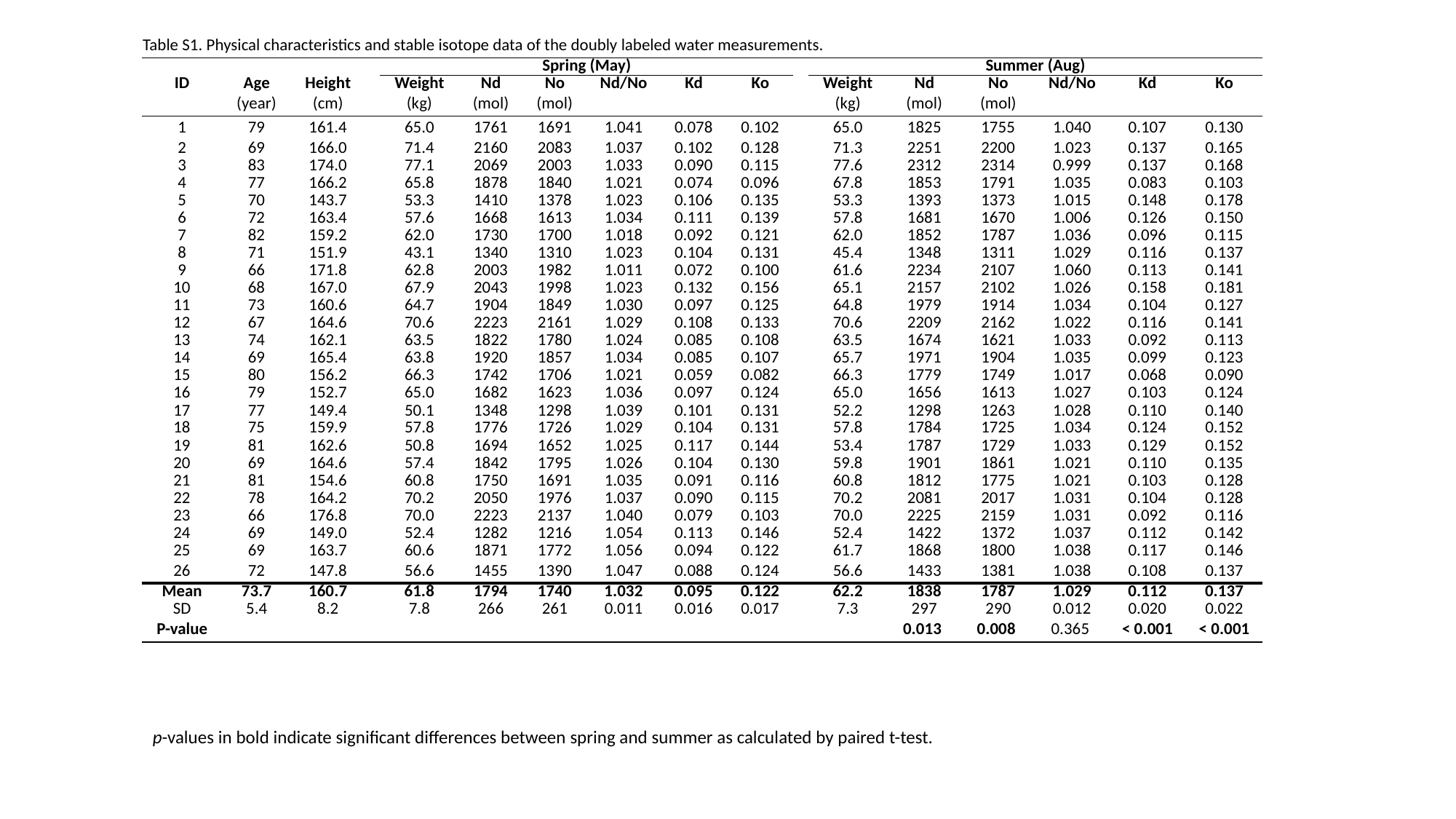

| Table S1. Physical characteristics and stable isotope data of the doubly labeled water measurements. | | | | | | | | | | | | | | | | |
| --- | --- | --- | --- | --- | --- | --- | --- | --- | --- | --- | --- | --- | --- | --- | --- | --- |
| | | | | Spring (May) | | | | | | | Summer (Aug) | | | | | |
| ID | Age | Height | | Weight | Nd | No | Nd/No | Kd | Ko | | Weight | Nd | No | Nd/No | Kd | Ko |
| | (year) | (cm) | | (kg) | (mol) | (mol) | | | | | (kg) | (mol) | (mol) | | | |
| 1 | 79 | 161.4 | | 65.0 | 1761 | 1691 | 1.041 | 0.078 | 0.102 | | 65.0 | 1825 | 1755 | 1.040 | 0.107 | 0.130 |
| 2 | 69 | 166.0 | | 71.4 | 2160 | 2083 | 1.037 | 0.102 | 0.128 | | 71.3 | 2251 | 2200 | 1.023 | 0.137 | 0.165 |
| 3 | 83 | 174.0 | | 77.1 | 2069 | 2003 | 1.033 | 0.090 | 0.115 | | 77.6 | 2312 | 2314 | 0.999 | 0.137 | 0.168 |
| 4 | 77 | 166.2 | | 65.8 | 1878 | 1840 | 1.021 | 0.074 | 0.096 | | 67.8 | 1853 | 1791 | 1.035 | 0.083 | 0.103 |
| 5 | 70 | 143.7 | | 53.3 | 1410 | 1378 | 1.023 | 0.106 | 0.135 | | 53.3 | 1393 | 1373 | 1.015 | 0.148 | 0.178 |
| 6 | 72 | 163.4 | | 57.6 | 1668 | 1613 | 1.034 | 0.111 | 0.139 | | 57.8 | 1681 | 1670 | 1.006 | 0.126 | 0.150 |
| 7 | 82 | 159.2 | | 62.0 | 1730 | 1700 | 1.018 | 0.092 | 0.121 | | 62.0 | 1852 | 1787 | 1.036 | 0.096 | 0.115 |
| 8 | 71 | 151.9 | | 43.1 | 1340 | 1310 | 1.023 | 0.104 | 0.131 | | 45.4 | 1348 | 1311 | 1.029 | 0.116 | 0.137 |
| 9 | 66 | 171.8 | | 62.8 | 2003 | 1982 | 1.011 | 0.072 | 0.100 | | 61.6 | 2234 | 2107 | 1.060 | 0.113 | 0.141 |
| 10 | 68 | 167.0 | | 67.9 | 2043 | 1998 | 1.023 | 0.132 | 0.156 | | 65.1 | 2157 | 2102 | 1.026 | 0.158 | 0.181 |
| 11 | 73 | 160.6 | | 64.7 | 1904 | 1849 | 1.030 | 0.097 | 0.125 | | 64.8 | 1979 | 1914 | 1.034 | 0.104 | 0.127 |
| 12 | 67 | 164.6 | | 70.6 | 2223 | 2161 | 1.029 | 0.108 | 0.133 | | 70.6 | 2209 | 2162 | 1.022 | 0.116 | 0.141 |
| 13 | 74 | 162.1 | | 63.5 | 1822 | 1780 | 1.024 | 0.085 | 0.108 | | 63.5 | 1674 | 1621 | 1.033 | 0.092 | 0.113 |
| 14 | 69 | 165.4 | | 63.8 | 1920 | 1857 | 1.034 | 0.085 | 0.107 | | 65.7 | 1971 | 1904 | 1.035 | 0.099 | 0.123 |
| 15 | 80 | 156.2 | | 66.3 | 1742 | 1706 | 1.021 | 0.059 | 0.082 | | 66.3 | 1779 | 1749 | 1.017 | 0.068 | 0.090 |
| 16 | 79 | 152.7 | | 65.0 | 1682 | 1623 | 1.036 | 0.097 | 0.124 | | 65.0 | 1656 | 1613 | 1.027 | 0.103 | 0.124 |
| 17 | 77 | 149.4 | | 50.1 | 1348 | 1298 | 1.039 | 0.101 | 0.131 | | 52.2 | 1298 | 1263 | 1.028 | 0.110 | 0.140 |
| 18 | 75 | 159.9 | | 57.8 | 1776 | 1726 | 1.029 | 0.104 | 0.131 | | 57.8 | 1784 | 1725 | 1.034 | 0.124 | 0.152 |
| 19 | 81 | 162.6 | | 50.8 | 1694 | 1652 | 1.025 | 0.117 | 0.144 | | 53.4 | 1787 | 1729 | 1.033 | 0.129 | 0.152 |
| 20 | 69 | 164.6 | | 57.4 | 1842 | 1795 | 1.026 | 0.104 | 0.130 | | 59.8 | 1901 | 1861 | 1.021 | 0.110 | 0.135 |
| 21 | 81 | 154.6 | | 60.8 | 1750 | 1691 | 1.035 | 0.091 | 0.116 | | 60.8 | 1812 | 1775 | 1.021 | 0.103 | 0.128 |
| 22 | 78 | 164.2 | | 70.2 | 2050 | 1976 | 1.037 | 0.090 | 0.115 | | 70.2 | 2081 | 2017 | 1.031 | 0.104 | 0.128 |
| 23 | 66 | 176.8 | | 70.0 | 2223 | 2137 | 1.040 | 0.079 | 0.103 | | 70.0 | 2225 | 2159 | 1.031 | 0.092 | 0.116 |
| 24 | 69 | 149.0 | | 52.4 | 1282 | 1216 | 1.054 | 0.113 | 0.146 | | 52.4 | 1422 | 1372 | 1.037 | 0.112 | 0.142 |
| 25 | 69 | 163.7 | | 60.6 | 1871 | 1772 | 1.056 | 0.094 | 0.122 | | 61.7 | 1868 | 1800 | 1.038 | 0.117 | 0.146 |
| 26 | 72 | 147.8 | | 56.6 | 1455 | 1390 | 1.047 | 0.088 | 0.124 | | 56.6 | 1433 | 1381 | 1.038 | 0.108 | 0.137 |
| Mean | 73.7 | 160.7 | | 61.8 | 1794 | 1740 | 1.032 | 0.095 | 0.122 | | 62.2 | 1838 | 1787 | 1.029 | 0.112 | 0.137 |
| SD | 5.4 | 8.2 | | 7.8 | 266 | 261 | 0.011 | 0.016 | 0.017 | | 7.3 | 297 | 290 | 0.012 | 0.020 | 0.022 |
| P-value | | | | | | | | | | | | 0.013 | 0.008 | 0.365 | < 0.001 | < 0.001 |
p-values in bold indicate significant differences between spring and summer as calculated by paired t-test.

## Slide 2
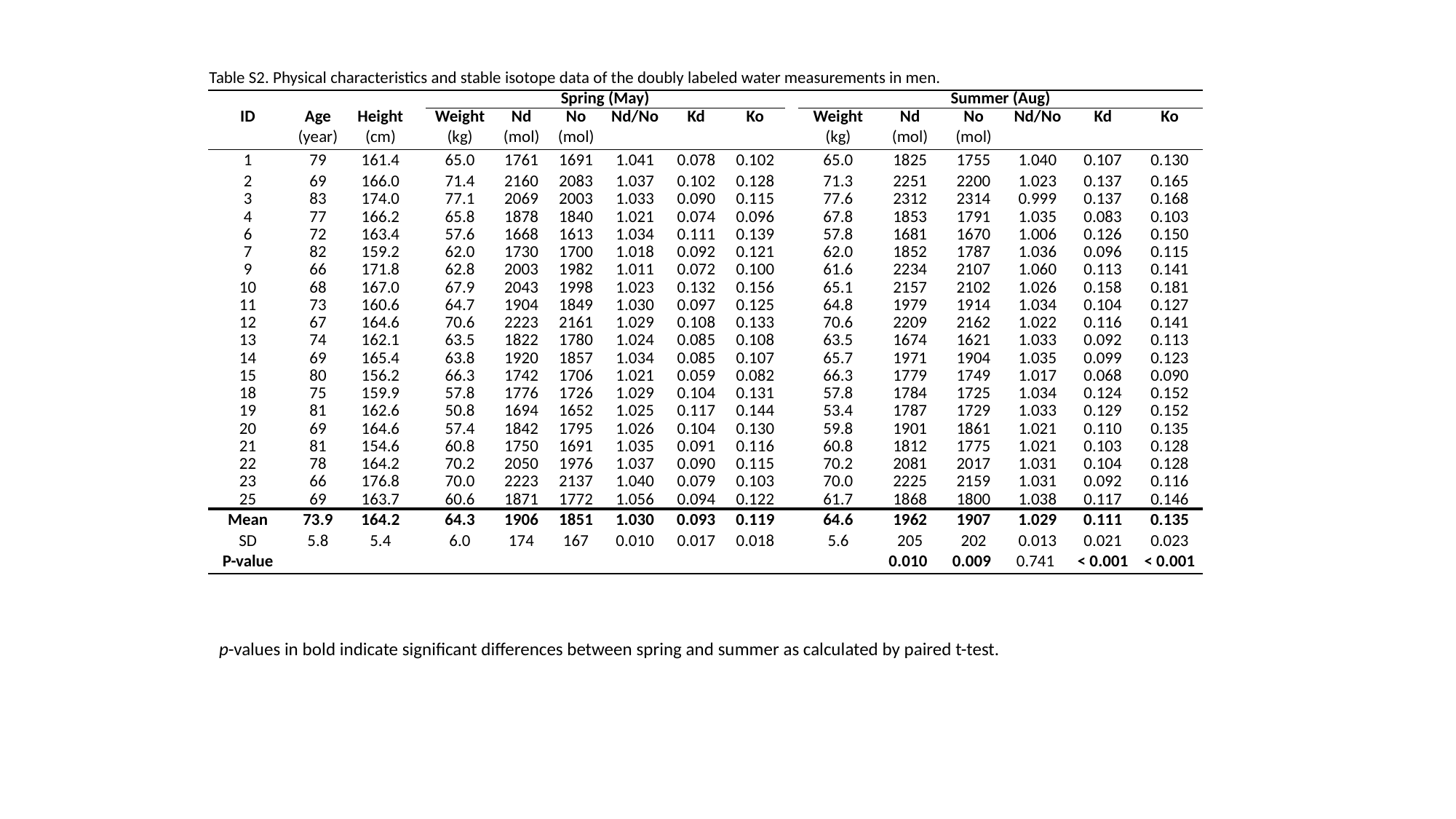

| Table S2. Physical characteristics and stable isotope data of the doubly labeled water measurements in men. | | | | | | | | | | | | | | | | |
| --- | --- | --- | --- | --- | --- | --- | --- | --- | --- | --- | --- | --- | --- | --- | --- | --- |
| | | | | Spring (May) | | | | | | | Summer (Aug) | | | | | |
| ID | Age | Height | | Weight | Nd | No | Nd/No | Kd | Ko | | Weight | Nd | No | Nd/No | Kd | Ko |
| | (year) | (cm) | | (kg) | (mol) | (mol) | | | | | (kg) | (mol) | (mol) | | | |
| 1 | 79 | 161.4 | | 65.0 | 1761 | 1691 | 1.041 | 0.078 | 0.102 | | 65.0 | 1825 | 1755 | 1.040 | 0.107 | 0.130 |
| 2 | 69 | 166.0 | | 71.4 | 2160 | 2083 | 1.037 | 0.102 | 0.128 | | 71.3 | 2251 | 2200 | 1.023 | 0.137 | 0.165 |
| 3 | 83 | 174.0 | | 77.1 | 2069 | 2003 | 1.033 | 0.090 | 0.115 | | 77.6 | 2312 | 2314 | 0.999 | 0.137 | 0.168 |
| 4 | 77 | 166.2 | | 65.8 | 1878 | 1840 | 1.021 | 0.074 | 0.096 | | 67.8 | 1853 | 1791 | 1.035 | 0.083 | 0.103 |
| 6 | 72 | 163.4 | | 57.6 | 1668 | 1613 | 1.034 | 0.111 | 0.139 | | 57.8 | 1681 | 1670 | 1.006 | 0.126 | 0.150 |
| 7 | 82 | 159.2 | | 62.0 | 1730 | 1700 | 1.018 | 0.092 | 0.121 | | 62.0 | 1852 | 1787 | 1.036 | 0.096 | 0.115 |
| 9 | 66 | 171.8 | | 62.8 | 2003 | 1982 | 1.011 | 0.072 | 0.100 | | 61.6 | 2234 | 2107 | 1.060 | 0.113 | 0.141 |
| 10 | 68 | 167.0 | | 67.9 | 2043 | 1998 | 1.023 | 0.132 | 0.156 | | 65.1 | 2157 | 2102 | 1.026 | 0.158 | 0.181 |
| 11 | 73 | 160.6 | | 64.7 | 1904 | 1849 | 1.030 | 0.097 | 0.125 | | 64.8 | 1979 | 1914 | 1.034 | 0.104 | 0.127 |
| 12 | 67 | 164.6 | | 70.6 | 2223 | 2161 | 1.029 | 0.108 | 0.133 | | 70.6 | 2209 | 2162 | 1.022 | 0.116 | 0.141 |
| 13 | 74 | 162.1 | | 63.5 | 1822 | 1780 | 1.024 | 0.085 | 0.108 | | 63.5 | 1674 | 1621 | 1.033 | 0.092 | 0.113 |
| 14 | 69 | 165.4 | | 63.8 | 1920 | 1857 | 1.034 | 0.085 | 0.107 | | 65.7 | 1971 | 1904 | 1.035 | 0.099 | 0.123 |
| 15 | 80 | 156.2 | | 66.3 | 1742 | 1706 | 1.021 | 0.059 | 0.082 | | 66.3 | 1779 | 1749 | 1.017 | 0.068 | 0.090 |
| 18 | 75 | 159.9 | | 57.8 | 1776 | 1726 | 1.029 | 0.104 | 0.131 | | 57.8 | 1784 | 1725 | 1.034 | 0.124 | 0.152 |
| 19 | 81 | 162.6 | | 50.8 | 1694 | 1652 | 1.025 | 0.117 | 0.144 | | 53.4 | 1787 | 1729 | 1.033 | 0.129 | 0.152 |
| 20 | 69 | 164.6 | | 57.4 | 1842 | 1795 | 1.026 | 0.104 | 0.130 | | 59.8 | 1901 | 1861 | 1.021 | 0.110 | 0.135 |
| 21 | 81 | 154.6 | | 60.8 | 1750 | 1691 | 1.035 | 0.091 | 0.116 | | 60.8 | 1812 | 1775 | 1.021 | 0.103 | 0.128 |
| 22 | 78 | 164.2 | | 70.2 | 2050 | 1976 | 1.037 | 0.090 | 0.115 | | 70.2 | 2081 | 2017 | 1.031 | 0.104 | 0.128 |
| 23 | 66 | 176.8 | | 70.0 | 2223 | 2137 | 1.040 | 0.079 | 0.103 | | 70.0 | 2225 | 2159 | 1.031 | 0.092 | 0.116 |
| 25 | 69 | 163.7 | | 60.6 | 1871 | 1772 | 1.056 | 0.094 | 0.122 | | 61.7 | 1868 | 1800 | 1.038 | 0.117 | 0.146 |
| Mean | 73.9 | 164.2 | | 64.3 | 1906 | 1851 | 1.030 | 0.093 | 0.119 | | 64.6 | 1962 | 1907 | 1.029 | 0.111 | 0.135 |
| SD | 5.8 | 5.4 | | 6.0 | 174 | 167 | 0.010 | 0.017 | 0.018 | | 5.6 | 205 | 202 | 0.013 | 0.021 | 0.023 |
| P-value | | | | | | | | | | | | 0.010 | 0.009 | 0.741 | < 0.001 | < 0.001 |
p-values in bold indicate significant differences between spring and summer as calculated by paired t-test.

## Slide 3
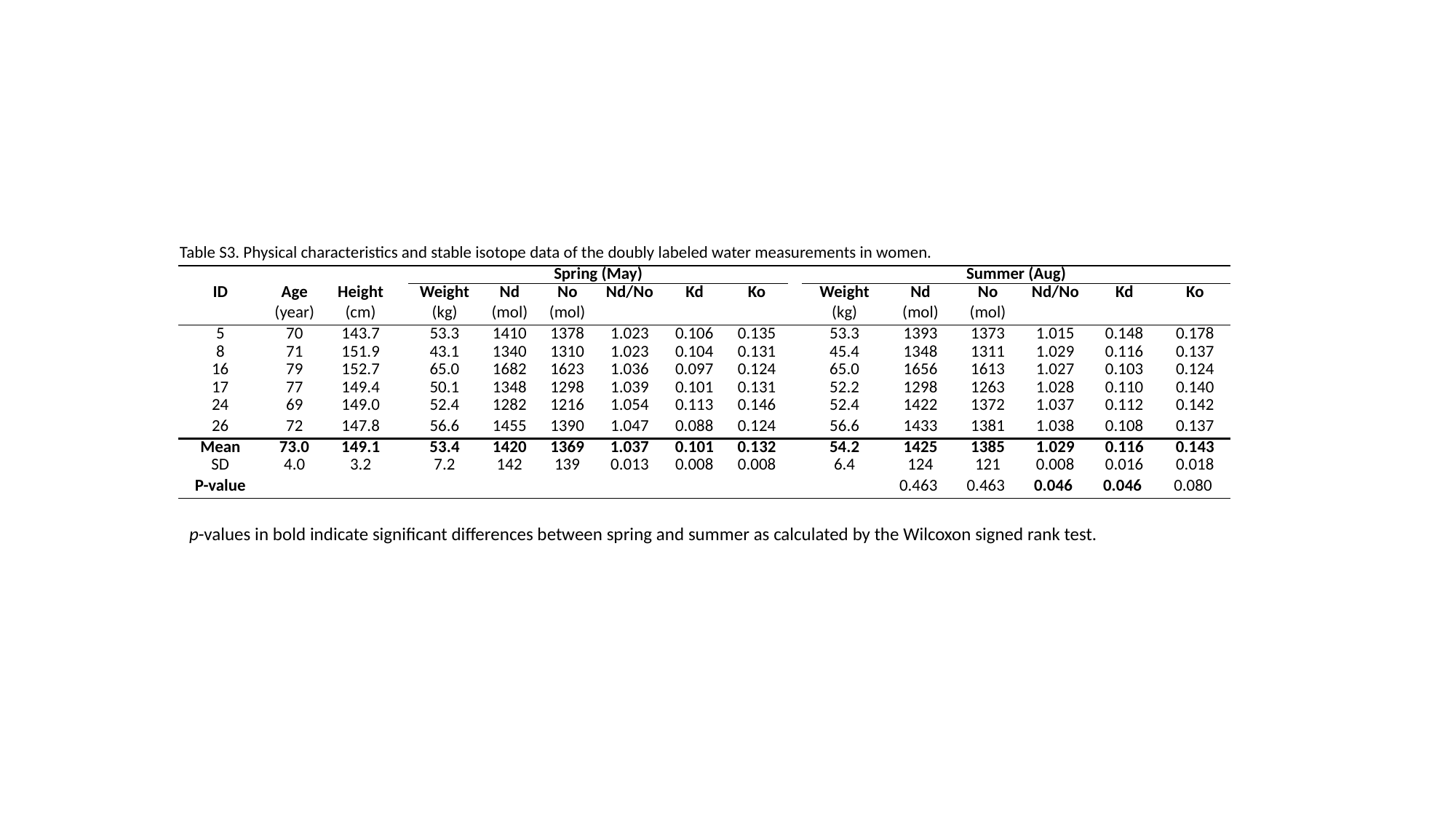

| Table S3. Physical characteristics and stable isotope data of the doubly labeled water measurements in women. | | | | | | | | | | | | | | | | |
| --- | --- | --- | --- | --- | --- | --- | --- | --- | --- | --- | --- | --- | --- | --- | --- | --- |
| | | | | Spring (May) | | | | | | | Summer (Aug) | | | | | |
| ID | Age | Height | | Weight | Nd | No | Nd/No | Kd | Ko | | Weight | Nd | No | Nd/No | Kd | Ko |
| | (year) | (cm) | | (kg) | (mol) | (mol) | | | | | (kg) | (mol) | (mol) | | | |
| 5 | 70 | 143.7 | | 53.3 | 1410 | 1378 | 1.023 | 0.106 | 0.135 | | 53.3 | 1393 | 1373 | 1.015 | 0.148 | 0.178 |
| 8 | 71 | 151.9 | | 43.1 | 1340 | 1310 | 1.023 | 0.104 | 0.131 | | 45.4 | 1348 | 1311 | 1.029 | 0.116 | 0.137 |
| 16 | 79 | 152.7 | | 65.0 | 1682 | 1623 | 1.036 | 0.097 | 0.124 | | 65.0 | 1656 | 1613 | 1.027 | 0.103 | 0.124 |
| 17 | 77 | 149.4 | | 50.1 | 1348 | 1298 | 1.039 | 0.101 | 0.131 | | 52.2 | 1298 | 1263 | 1.028 | 0.110 | 0.140 |
| 24 | 69 | 149.0 | | 52.4 | 1282 | 1216 | 1.054 | 0.113 | 0.146 | | 52.4 | 1422 | 1372 | 1.037 | 0.112 | 0.142 |
| 26 | 72 | 147.8 | | 56.6 | 1455 | 1390 | 1.047 | 0.088 | 0.124 | | 56.6 | 1433 | 1381 | 1.038 | 0.108 | 0.137 |
| Mean | 73.0 | 149.1 | | 53.4 | 1420 | 1369 | 1.037 | 0.101 | 0.132 | | 54.2 | 1425 | 1385 | 1.029 | 0.116 | 0.143 |
| SD | 4.0 | 3.2 | | 7.2 | 142 | 139 | 0.013 | 0.008 | 0.008 | | 6.4 | 124 | 121 | 0.008 | 0.016 | 0.018 |
| P-value | | | | | | | | | | | | 0.463 | 0.463 | 0.046 | 0.046 | 0.080 |
p-values in bold indicate significant differences between spring and summer as calculated by the Wilcoxon signed rank test.

## Slide 4
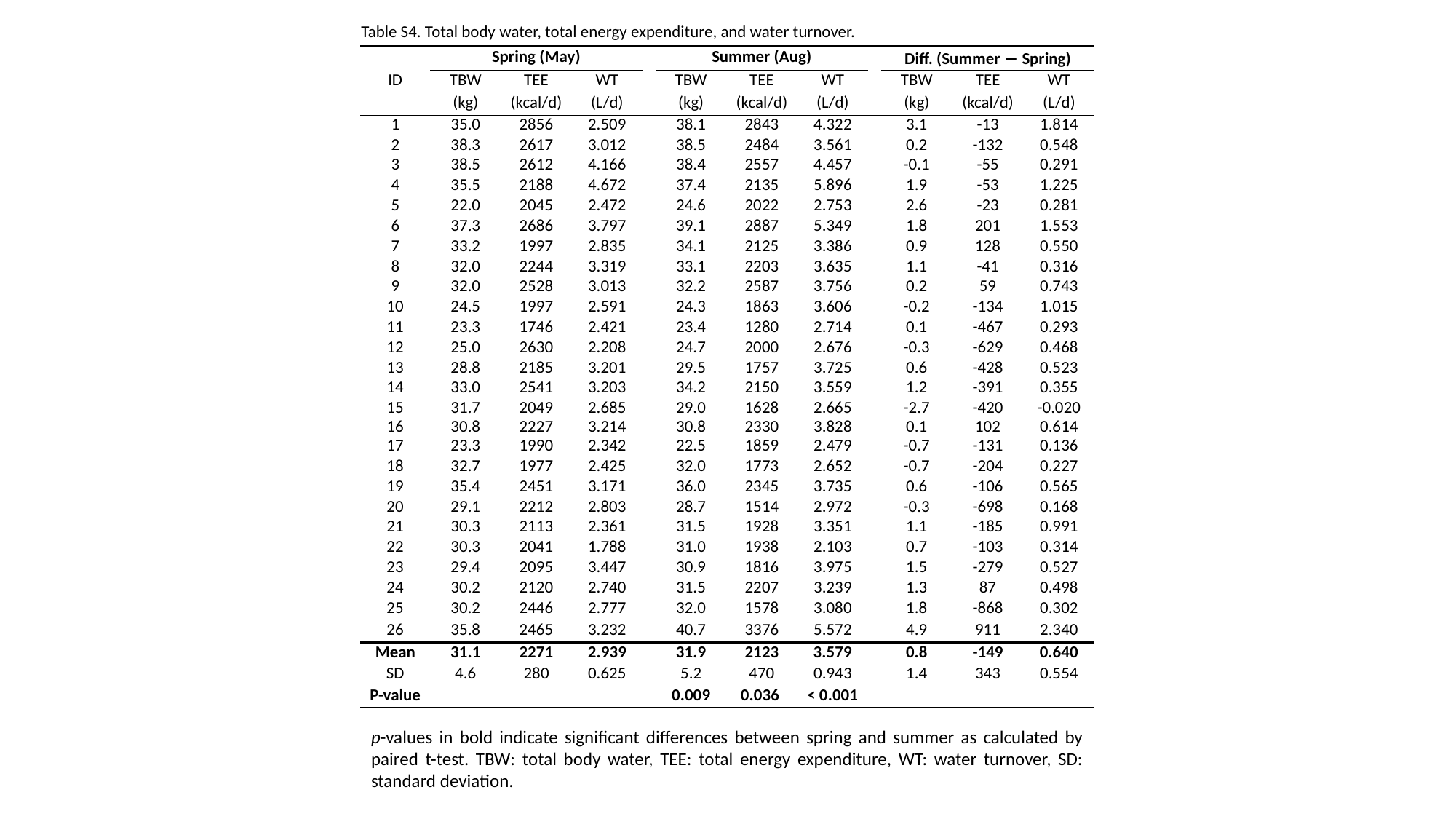

| Table S4. Total body water, total energy expenditure, and water turnover. | | | | | | | | | | | |
| --- | --- | --- | --- | --- | --- | --- | --- | --- | --- | --- | --- |
| | Spring (May) | | | | Summer (Aug) | | | | Diff. (Summer − Spring) | | |
| ID | TBW | TEE | WT | | TBW | TEE | WT | | TBW | TEE | WT |
| | (kg) | (kcal/d) | (L/d) | | (kg) | (kcal/d) | (L/d) | | (kg) | (kcal/d) | (L/d) |
| 1 | 35.0 | 2856 | 2.509 | | 38.1 | 2843 | 4.322 | | 3.1 | -13 | 1.814 |
| 2 | 38.3 | 2617 | 3.012 | | 38.5 | 2484 | 3.561 | | 0.2 | -132 | 0.548 |
| 3 | 38.5 | 2612 | 4.166 | | 38.4 | 2557 | 4.457 | | -0.1 | -55 | 0.291 |
| 4 | 35.5 | 2188 | 4.672 | | 37.4 | 2135 | 5.896 | | 1.9 | -53 | 1.225 |
| 5 | 22.0 | 2045 | 2.472 | | 24.6 | 2022 | 2.753 | | 2.6 | -23 | 0.281 |
| 6 | 37.3 | 2686 | 3.797 | | 39.1 | 2887 | 5.349 | | 1.8 | 201 | 1.553 |
| 7 | 33.2 | 1997 | 2.835 | | 34.1 | 2125 | 3.386 | | 0.9 | 128 | 0.550 |
| 8 | 32.0 | 2244 | 3.319 | | 33.1 | 2203 | 3.635 | | 1.1 | -41 | 0.316 |
| 9 | 32.0 | 2528 | 3.013 | | 32.2 | 2587 | 3.756 | | 0.2 | 59 | 0.743 |
| 10 | 24.5 | 1997 | 2.591 | | 24.3 | 1863 | 3.606 | | -0.2 | -134 | 1.015 |
| 11 | 23.3 | 1746 | 2.421 | | 23.4 | 1280 | 2.714 | | 0.1 | -467 | 0.293 |
| 12 | 25.0 | 2630 | 2.208 | | 24.7 | 2000 | 2.676 | | -0.3 | -629 | 0.468 |
| 13 | 28.8 | 2185 | 3.201 | | 29.5 | 1757 | 3.725 | | 0.6 | -428 | 0.523 |
| 14 | 33.0 | 2541 | 3.203 | | 34.2 | 2150 | 3.559 | | 1.2 | -391 | 0.355 |
| 15 | 31.7 | 2049 | 2.685 | | 29.0 | 1628 | 2.665 | | -2.7 | -420 | -0.020 |
| 16 | 30.8 | 2227 | 3.214 | | 30.8 | 2330 | 3.828 | | 0.1 | 102 | 0.614 |
| 17 | 23.3 | 1990 | 2.342 | | 22.5 | 1859 | 2.479 | | -0.7 | -131 | 0.136 |
| 18 | 32.7 | 1977 | 2.425 | | 32.0 | 1773 | 2.652 | | -0.7 | -204 | 0.227 |
| 19 | 35.4 | 2451 | 3.171 | | 36.0 | 2345 | 3.735 | | 0.6 | -106 | 0.565 |
| 20 | 29.1 | 2212 | 2.803 | | 28.7 | 1514 | 2.972 | | -0.3 | -698 | 0.168 |
| 21 | 30.3 | 2113 | 2.361 | | 31.5 | 1928 | 3.351 | | 1.1 | -185 | 0.991 |
| 22 | 30.3 | 2041 | 1.788 | | 31.0 | 1938 | 2.103 | | 0.7 | -103 | 0.314 |
| 23 | 29.4 | 2095 | 3.447 | | 30.9 | 1816 | 3.975 | | 1.5 | -279 | 0.527 |
| 24 | 30.2 | 2120 | 2.740 | | 31.5 | 2207 | 3.239 | | 1.3 | 87 | 0.498 |
| 25 | 30.2 | 2446 | 2.777 | | 32.0 | 1578 | 3.080 | | 1.8 | -868 | 0.302 |
| 26 | 35.8 | 2465 | 3.232 | | 40.7 | 3376 | 5.572 | | 4.9 | 911 | 2.340 |
| Mean | 31.1 | 2271 | 2.939 | | 31.9 | 2123 | 3.579 | | 0.8 | -149 | 0.640 |
| SD | 4.6 | 280 | 0.625 | | 5.2 | 470 | 0.943 | | 1.4 | 343 | 0.554 |
| P-value | | | | | 0.009 | 0.036 | < 0.001 | | | | |
p-values in bold indicate significant differences between spring and summer as calculated by paired t-test. TBW: total body water, TEE: total energy expenditure, WT: water turnover, SD: standard deviation.

## Slide 5
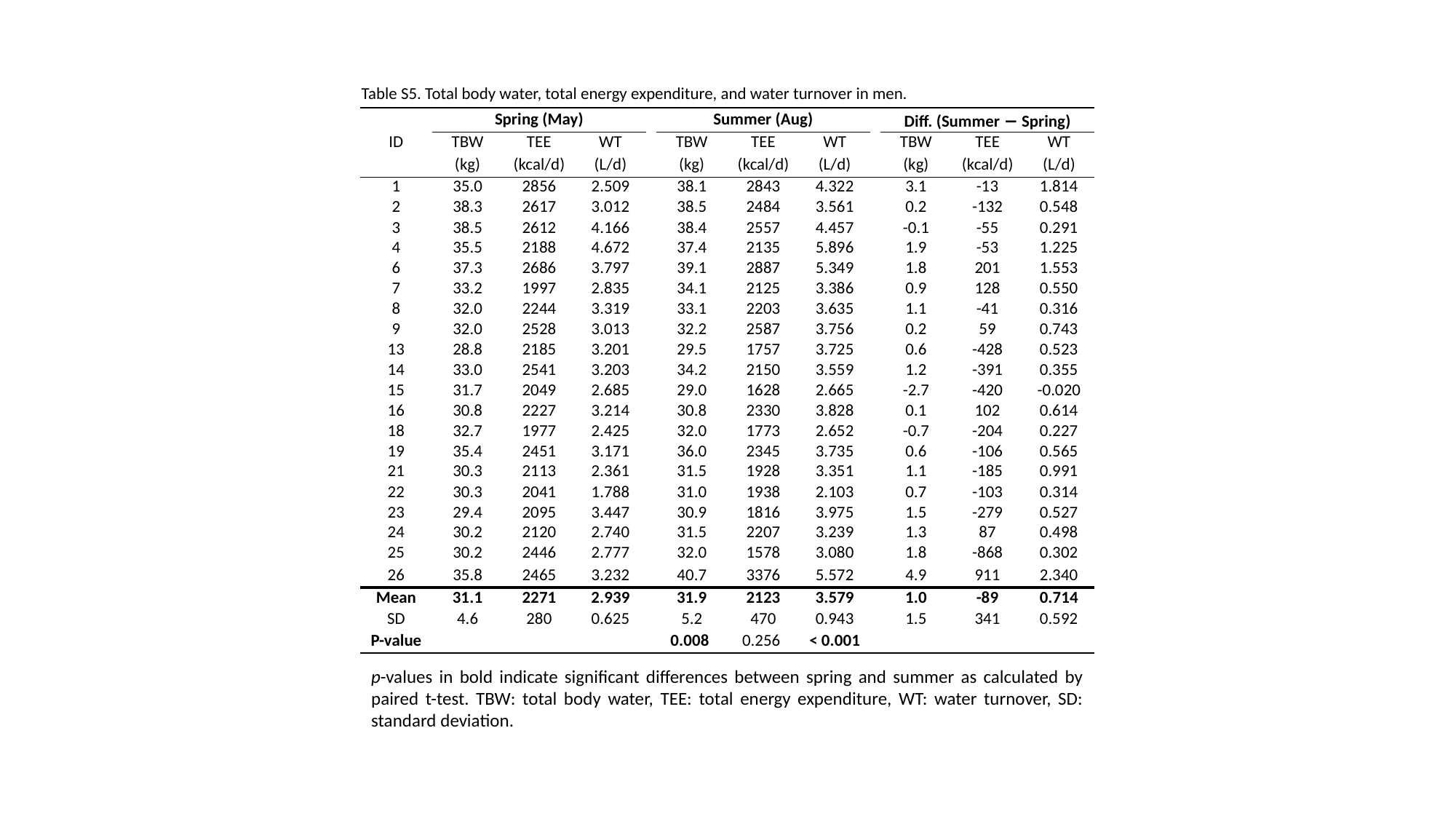

| Table S5. Total body water, total energy expenditure, and water turnover in men. | | | | | | | | | | | |
| --- | --- | --- | --- | --- | --- | --- | --- | --- | --- | --- | --- |
| | Spring (May) | | | | Summer (Aug) | | | | Diff. (Summer − Spring) | | |
| ID | TBW | TEE | WT | | TBW | TEE | WT | | TBW | TEE | WT |
| | (kg) | (kcal/d) | (L/d) | | (kg) | (kcal/d) | (L/d) | | (kg) | (kcal/d) | (L/d) |
| 1 | 35.0 | 2856 | 2.509 | | 38.1 | 2843 | 4.322 | | 3.1 | -13 | 1.814 |
| 2 | 38.3 | 2617 | 3.012 | | 38.5 | 2484 | 3.561 | | 0.2 | -132 | 0.548 |
| 3 | 38.5 | 2612 | 4.166 | | 38.4 | 2557 | 4.457 | | -0.1 | -55 | 0.291 |
| 4 | 35.5 | 2188 | 4.672 | | 37.4 | 2135 | 5.896 | | 1.9 | -53 | 1.225 |
| 6 | 37.3 | 2686 | 3.797 | | 39.1 | 2887 | 5.349 | | 1.8 | 201 | 1.553 |
| 7 | 33.2 | 1997 | 2.835 | | 34.1 | 2125 | 3.386 | | 0.9 | 128 | 0.550 |
| 8 | 32.0 | 2244 | 3.319 | | 33.1 | 2203 | 3.635 | | 1.1 | -41 | 0.316 |
| 9 | 32.0 | 2528 | 3.013 | | 32.2 | 2587 | 3.756 | | 0.2 | 59 | 0.743 |
| 13 | 28.8 | 2185 | 3.201 | | 29.5 | 1757 | 3.725 | | 0.6 | -428 | 0.523 |
| 14 | 33.0 | 2541 | 3.203 | | 34.2 | 2150 | 3.559 | | 1.2 | -391 | 0.355 |
| 15 | 31.7 | 2049 | 2.685 | | 29.0 | 1628 | 2.665 | | -2.7 | -420 | -0.020 |
| 16 | 30.8 | 2227 | 3.214 | | 30.8 | 2330 | 3.828 | | 0.1 | 102 | 0.614 |
| 18 | 32.7 | 1977 | 2.425 | | 32.0 | 1773 | 2.652 | | -0.7 | -204 | 0.227 |
| 19 | 35.4 | 2451 | 3.171 | | 36.0 | 2345 | 3.735 | | 0.6 | -106 | 0.565 |
| 21 | 30.3 | 2113 | 2.361 | | 31.5 | 1928 | 3.351 | | 1.1 | -185 | 0.991 |
| 22 | 30.3 | 2041 | 1.788 | | 31.0 | 1938 | 2.103 | | 0.7 | -103 | 0.314 |
| 23 | 29.4 | 2095 | 3.447 | | 30.9 | 1816 | 3.975 | | 1.5 | -279 | 0.527 |
| 24 | 30.2 | 2120 | 2.740 | | 31.5 | 2207 | 3.239 | | 1.3 | 87 | 0.498 |
| 25 | 30.2 | 2446 | 2.777 | | 32.0 | 1578 | 3.080 | | 1.8 | -868 | 0.302 |
| 26 | 35.8 | 2465 | 3.232 | | 40.7 | 3376 | 5.572 | | 4.9 | 911 | 2.340 |
| Mean | 31.1 | 2271 | 2.939 | | 31.9 | 2123 | 3.579 | | 1.0 | -89 | 0.714 |
| SD | 4.6 | 280 | 0.625 | | 5.2 | 470 | 0.943 | | 1.5 | 341 | 0.592 |
| P-value | | | | | 0.008 | 0.256 | < 0.001 | | | | |
p-values in bold indicate significant differences between spring and summer as calculated by paired t-test. TBW: total body water, TEE: total energy expenditure, WT: water turnover, SD: standard deviation.

## Slide 6
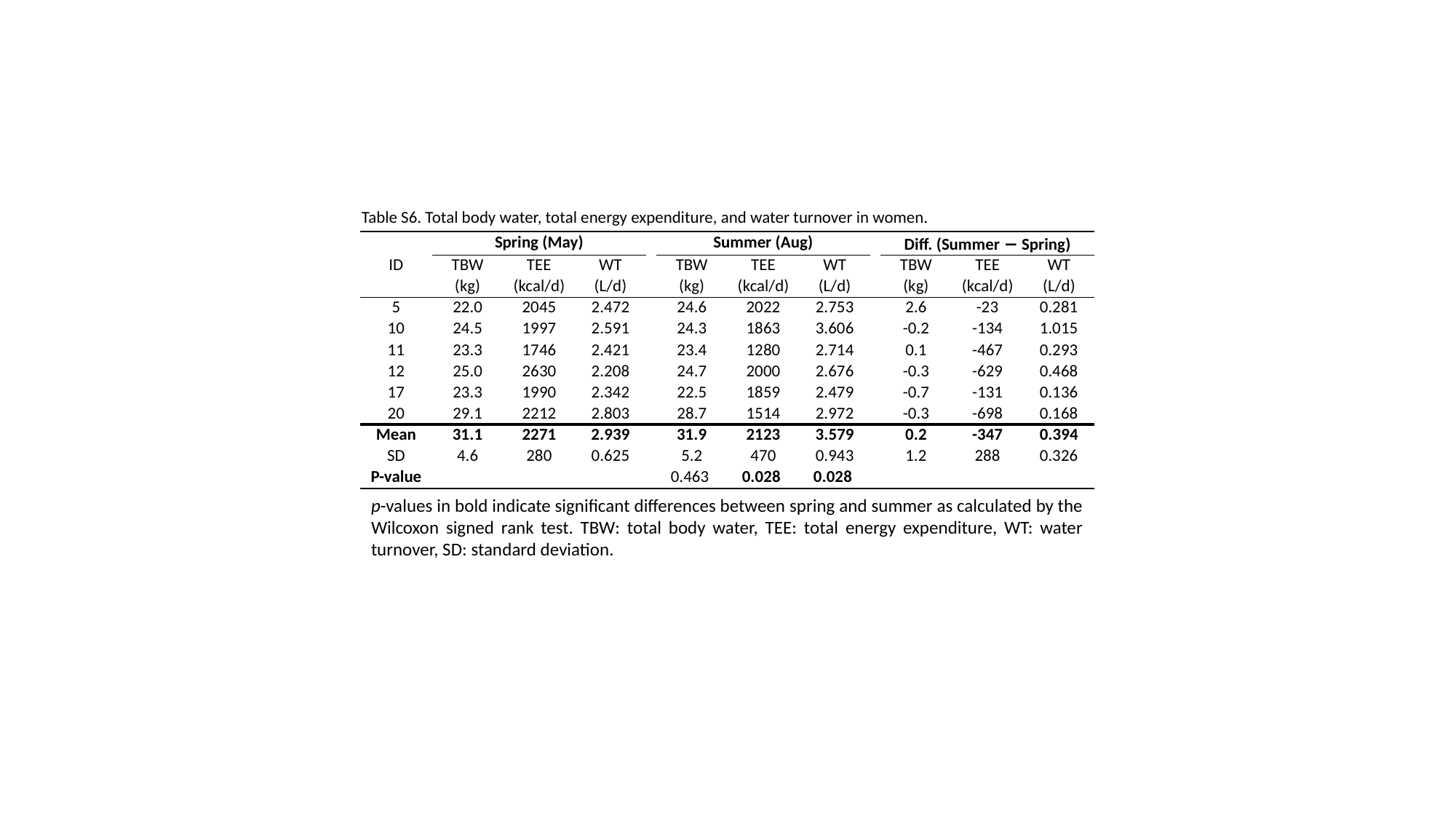

| Table S6. Total body water, total energy expenditure, and water turnover in women. | | | | | | | | | | | |
| --- | --- | --- | --- | --- | --- | --- | --- | --- | --- | --- | --- |
| | Spring (May) | | | | Summer (Aug) | | | | Diff. (Summer − Spring) | | |
| ID | TBW | TEE | WT | | TBW | TEE | WT | | TBW | TEE | WT |
| | (kg) | (kcal/d) | (L/d) | | (kg) | (kcal/d) | (L/d) | | (kg) | (kcal/d) | (L/d) |
| 5 | 22.0 | 2045 | 2.472 | | 24.6 | 2022 | 2.753 | | 2.6 | -23 | 0.281 |
| 10 | 24.5 | 1997 | 2.591 | | 24.3 | 1863 | 3.606 | | -0.2 | -134 | 1.015 |
| 11 | 23.3 | 1746 | 2.421 | | 23.4 | 1280 | 2.714 | | 0.1 | -467 | 0.293 |
| 12 | 25.0 | 2630 | 2.208 | | 24.7 | 2000 | 2.676 | | -0.3 | -629 | 0.468 |
| 17 | 23.3 | 1990 | 2.342 | | 22.5 | 1859 | 2.479 | | -0.7 | -131 | 0.136 |
| 20 | 29.1 | 2212 | 2.803 | | 28.7 | 1514 | 2.972 | | -0.3 | -698 | 0.168 |
| Mean | 31.1 | 2271 | 2.939 | | 31.9 | 2123 | 3.579 | | 0.2 | -347 | 0.394 |
| SD | 4.6 | 280 | 0.625 | | 5.2 | 470 | 0.943 | | 1.2 | 288 | 0.326 |
| P-value | | | | | 0.463 | 0.028 | 0.028 | | | | |
p-values in bold indicate significant differences between spring and summer as calculated by the Wilcoxon signed rank test. TBW: total body water, TEE: total energy expenditure, WT: water turnover, SD: standard deviation.

## Slide 7
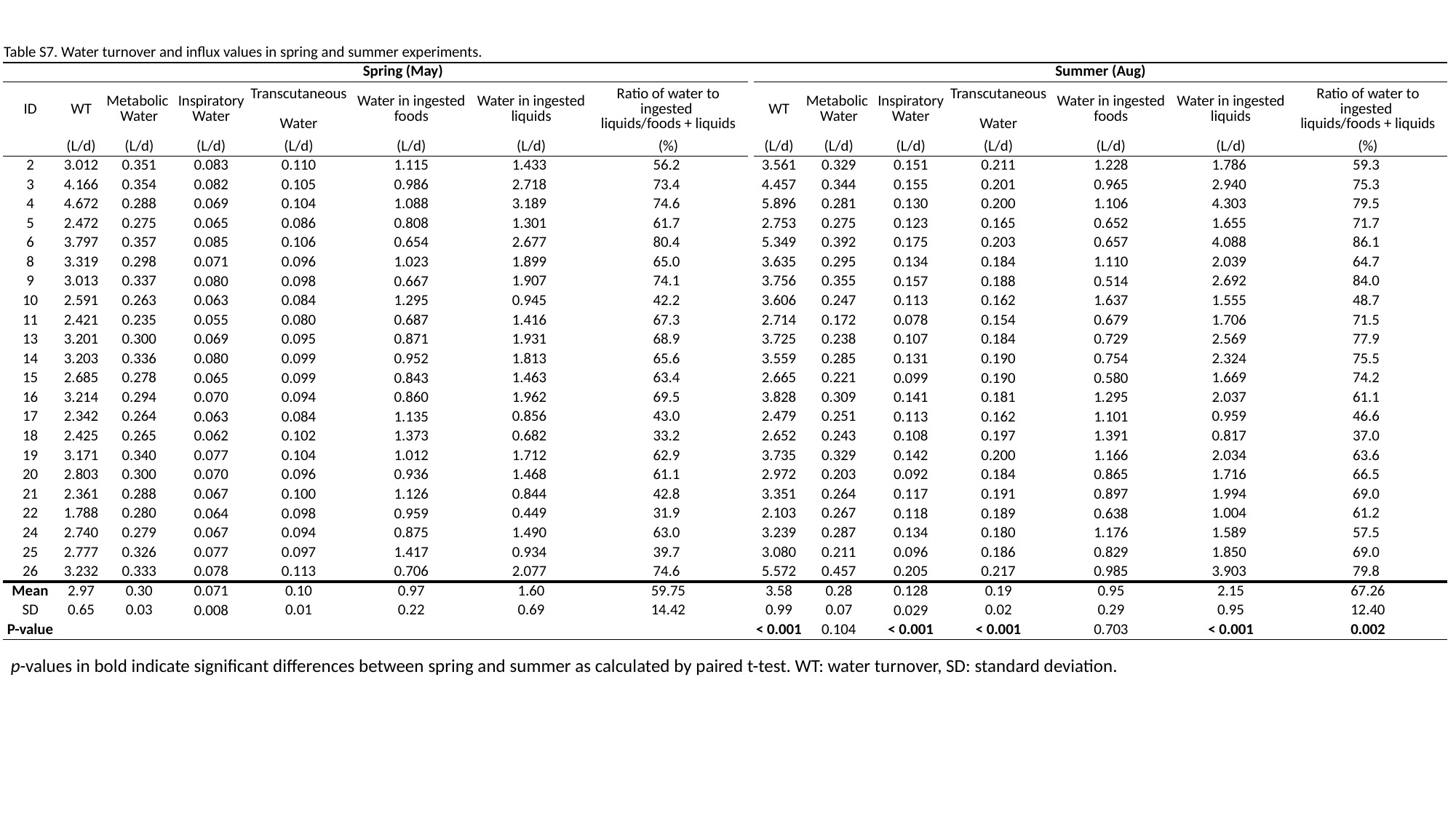

| Table S7. Water turnover and influx values in spring and summer experiments. | | | | | | | | | | | | | | | |
| --- | --- | --- | --- | --- | --- | --- | --- | --- | --- | --- | --- | --- | --- | --- | --- |
| | Spring (May) | | | | | | | | Summer (Aug) | | | | | | |
| ID | WT | Metabolic Water | Inspiratory Water | Transcutaneous Water | Water in ingested foods | Water in ingested liquids | Ratio of water to ingested liquids/foods + liquids | | WT | Metabolic Water | Inspiratory Water | Transcutaneous Water | Water in ingested foods | Water in ingested liquids | Ratio of water to ingested liquids/foods + liquids |
| | (L/d) | (L/d) | (L/d) | (L/d) | (L/d) | (L/d) | (%) | | (L/d) | (L/d) | (L/d) | (L/d) | (L/d) | (L/d) | (%) |
| 2 | 3.012 | 0.351 | 0.083 | 0.110 | 1.115 | 1.433 | 56.2 | | 3.561 | 0.329 | 0.151 | 0.211 | 1.228 | 1.786 | 59.3 |
| 3 | 4.166 | 0.354 | 0.082 | 0.105 | 0.986 | 2.718 | 73.4 | | 4.457 | 0.344 | 0.155 | 0.201 | 0.965 | 2.940 | 75.3 |
| 4 | 4.672 | 0.288 | 0.069 | 0.104 | 1.088 | 3.189 | 74.6 | | 5.896 | 0.281 | 0.130 | 0.200 | 1.106 | 4.303 | 79.5 |
| 5 | 2.472 | 0.275 | 0.065 | 0.086 | 0.808 | 1.301 | 61.7 | | 2.753 | 0.275 | 0.123 | 0.165 | 0.652 | 1.655 | 71.7 |
| 6 | 3.797 | 0.357 | 0.085 | 0.106 | 0.654 | 2.677 | 80.4 | | 5.349 | 0.392 | 0.175 | 0.203 | 0.657 | 4.088 | 86.1 |
| 8 | 3.319 | 0.298 | 0.071 | 0.096 | 1.023 | 1.899 | 65.0 | | 3.635 | 0.295 | 0.134 | 0.184 | 1.110 | 2.039 | 64.7 |
| 9 | 3.013 | 0.337 | 0.080 | 0.098 | 0.667 | 1.907 | 74.1 | | 3.756 | 0.355 | 0.157 | 0.188 | 0.514 | 2.692 | 84.0 |
| 10 | 2.591 | 0.263 | 0.063 | 0.084 | 1.295 | 0.945 | 42.2 | | 3.606 | 0.247 | 0.113 | 0.162 | 1.637 | 1.555 | 48.7 |
| 11 | 2.421 | 0.235 | 0.055 | 0.080 | 0.687 | 1.416 | 67.3 | | 2.714 | 0.172 | 0.078 | 0.154 | 0.679 | 1.706 | 71.5 |
| 13 | 3.201 | 0.300 | 0.069 | 0.095 | 0.871 | 1.931 | 68.9 | | 3.725 | 0.238 | 0.107 | 0.184 | 0.729 | 2.569 | 77.9 |
| 14 | 3.203 | 0.336 | 0.080 | 0.099 | 0.952 | 1.813 | 65.6 | | 3.559 | 0.285 | 0.131 | 0.190 | 0.754 | 2.324 | 75.5 |
| 15 | 2.685 | 0.278 | 0.065 | 0.099 | 0.843 | 1.463 | 63.4 | | 2.665 | 0.221 | 0.099 | 0.190 | 0.580 | 1.669 | 74.2 |
| 16 | 3.214 | 0.294 | 0.070 | 0.094 | 0.860 | 1.962 | 69.5 | | 3.828 | 0.309 | 0.141 | 0.181 | 1.295 | 2.037 | 61.1 |
| 17 | 2.342 | 0.264 | 0.063 | 0.084 | 1.135 | 0.856 | 43.0 | | 2.479 | 0.251 | 0.113 | 0.162 | 1.101 | 0.959 | 46.6 |
| 18 | 2.425 | 0.265 | 0.062 | 0.102 | 1.373 | 0.682 | 33.2 | | 2.652 | 0.243 | 0.108 | 0.197 | 1.391 | 0.817 | 37.0 |
| 19 | 3.171 | 0.340 | 0.077 | 0.104 | 1.012 | 1.712 | 62.9 | | 3.735 | 0.329 | 0.142 | 0.200 | 1.166 | 2.034 | 63.6 |
| 20 | 2.803 | 0.300 | 0.070 | 0.096 | 0.936 | 1.468 | 61.1 | | 2.972 | 0.203 | 0.092 | 0.184 | 0.865 | 1.716 | 66.5 |
| 21 | 2.361 | 0.288 | 0.067 | 0.100 | 1.126 | 0.844 | 42.8 | | 3.351 | 0.264 | 0.117 | 0.191 | 0.897 | 1.994 | 69.0 |
| 22 | 1.788 | 0.280 | 0.064 | 0.098 | 0.959 | 0.449 | 31.9 | | 2.103 | 0.267 | 0.118 | 0.189 | 0.638 | 1.004 | 61.2 |
| 24 | 2.740 | 0.279 | 0.067 | 0.094 | 0.875 | 1.490 | 63.0 | | 3.239 | 0.287 | 0.134 | 0.180 | 1.176 | 1.589 | 57.5 |
| 25 | 2.777 | 0.326 | 0.077 | 0.097 | 1.417 | 0.934 | 39.7 | | 3.080 | 0.211 | 0.096 | 0.186 | 0.829 | 1.850 | 69.0 |
| 26 | 3.232 | 0.333 | 0.078 | 0.113 | 0.706 | 2.077 | 74.6 | | 5.572 | 0.457 | 0.205 | 0.217 | 0.985 | 3.903 | 79.8 |
| Mean | 2.97 | 0.30 | 0.071 | 0.10 | 0.97 | 1.60 | 59.75 | | 3.58 | 0.28 | 0.128 | 0.19 | 0.95 | 2.15 | 67.26 |
| SD | 0.65 | 0.03 | 0.008 | 0.01 | 0.22 | 0.69 | 14.42 | | 0.99 | 0.07 | 0.029 | 0.02 | 0.29 | 0.95 | 12.40 |
| P-value | | | | | | | | | < 0.001 | 0.104 | < 0.001 | < 0.001 | 0.703 | < 0.001 | 0.002 |
p-values in bold indicate significant differences between spring and summer as calculated by paired t-test. WT: water turnover, SD: standard deviation.

## Slide 8
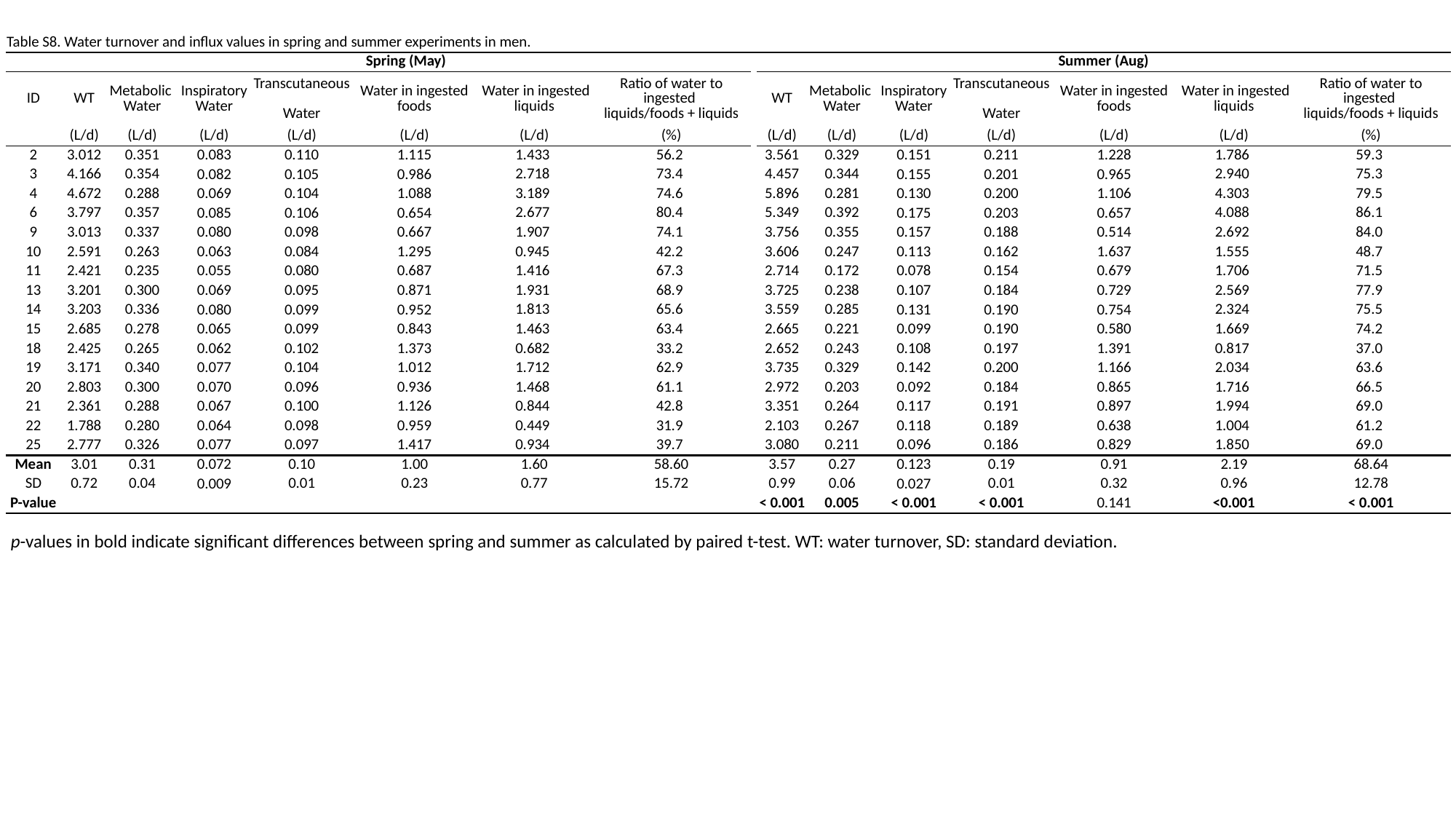

| Table S8. Water turnover and influx values in spring and summer experiments in men. | | | | | | | | | | | | | | | |
| --- | --- | --- | --- | --- | --- | --- | --- | --- | --- | --- | --- | --- | --- | --- | --- |
| | Spring (May) | | | | | | | | Summer (Aug) | | | | | | |
| ID | WT | Metabolic Water | Inspiratory Water | Transcutaneous Water | Water in ingested foods | Water in ingested liquids | Ratio of water to ingested liquids/foods + liquids | | WT | Metabolic Water | Inspiratory Water | Transcutaneous Water | Water in ingested foods | Water in ingested liquids | Ratio of water to ingested liquids/foods + liquids |
| | (L/d) | (L/d) | (L/d) | (L/d) | (L/d) | (L/d) | (%) | | (L/d) | (L/d) | (L/d) | (L/d) | (L/d) | (L/d) | (%) |
| 2 | 3.012 | 0.351 | 0.083 | 0.110 | 1.115 | 1.433 | 56.2 | | 3.561 | 0.329 | 0.151 | 0.211 | 1.228 | 1.786 | 59.3 |
| 3 | 4.166 | 0.354 | 0.082 | 0.105 | 0.986 | 2.718 | 73.4 | | 4.457 | 0.344 | 0.155 | 0.201 | 0.965 | 2.940 | 75.3 |
| 4 | 4.672 | 0.288 | 0.069 | 0.104 | 1.088 | 3.189 | 74.6 | | 5.896 | 0.281 | 0.130 | 0.200 | 1.106 | 4.303 | 79.5 |
| 6 | 3.797 | 0.357 | 0.085 | 0.106 | 0.654 | 2.677 | 80.4 | | 5.349 | 0.392 | 0.175 | 0.203 | 0.657 | 4.088 | 86.1 |
| 9 | 3.013 | 0.337 | 0.080 | 0.098 | 0.667 | 1.907 | 74.1 | | 3.756 | 0.355 | 0.157 | 0.188 | 0.514 | 2.692 | 84.0 |
| 10 | 2.591 | 0.263 | 0.063 | 0.084 | 1.295 | 0.945 | 42.2 | | 3.606 | 0.247 | 0.113 | 0.162 | 1.637 | 1.555 | 48.7 |
| 11 | 2.421 | 0.235 | 0.055 | 0.080 | 0.687 | 1.416 | 67.3 | | 2.714 | 0.172 | 0.078 | 0.154 | 0.679 | 1.706 | 71.5 |
| 13 | 3.201 | 0.300 | 0.069 | 0.095 | 0.871 | 1.931 | 68.9 | | 3.725 | 0.238 | 0.107 | 0.184 | 0.729 | 2.569 | 77.9 |
| 14 | 3.203 | 0.336 | 0.080 | 0.099 | 0.952 | 1.813 | 65.6 | | 3.559 | 0.285 | 0.131 | 0.190 | 0.754 | 2.324 | 75.5 |
| 15 | 2.685 | 0.278 | 0.065 | 0.099 | 0.843 | 1.463 | 63.4 | | 2.665 | 0.221 | 0.099 | 0.190 | 0.580 | 1.669 | 74.2 |
| 18 | 2.425 | 0.265 | 0.062 | 0.102 | 1.373 | 0.682 | 33.2 | | 2.652 | 0.243 | 0.108 | 0.197 | 1.391 | 0.817 | 37.0 |
| 19 | 3.171 | 0.340 | 0.077 | 0.104 | 1.012 | 1.712 | 62.9 | | 3.735 | 0.329 | 0.142 | 0.200 | 1.166 | 2.034 | 63.6 |
| 20 | 2.803 | 0.300 | 0.070 | 0.096 | 0.936 | 1.468 | 61.1 | | 2.972 | 0.203 | 0.092 | 0.184 | 0.865 | 1.716 | 66.5 |
| 21 | 2.361 | 0.288 | 0.067 | 0.100 | 1.126 | 0.844 | 42.8 | | 3.351 | 0.264 | 0.117 | 0.191 | 0.897 | 1.994 | 69.0 |
| 22 | 1.788 | 0.280 | 0.064 | 0.098 | 0.959 | 0.449 | 31.9 | | 2.103 | 0.267 | 0.118 | 0.189 | 0.638 | 1.004 | 61.2 |
| 25 | 2.777 | 0.326 | 0.077 | 0.097 | 1.417 | 0.934 | 39.7 | | 3.080 | 0.211 | 0.096 | 0.186 | 0.829 | 1.850 | 69.0 |
| Mean | 3.01 | 0.31 | 0.072 | 0.10 | 1.00 | 1.60 | 58.60 | | 3.57 | 0.27 | 0.123 | 0.19 | 0.91 | 2.19 | 68.64 |
| SD | 0.72 | 0.04 | 0.009 | 0.01 | 0.23 | 0.77 | 15.72 | | 0.99 | 0.06 | 0.027 | 0.01 | 0.32 | 0.96 | 12.78 |
| P-value | | | | | | | | | < 0.001 | 0.005 | < 0.001 | < 0.001 | 0.141 | <0.001 | < 0.001 |
p-values in bold indicate significant differences between spring and summer as calculated by paired t-test. WT: water turnover, SD: standard deviation.

## Slide 9
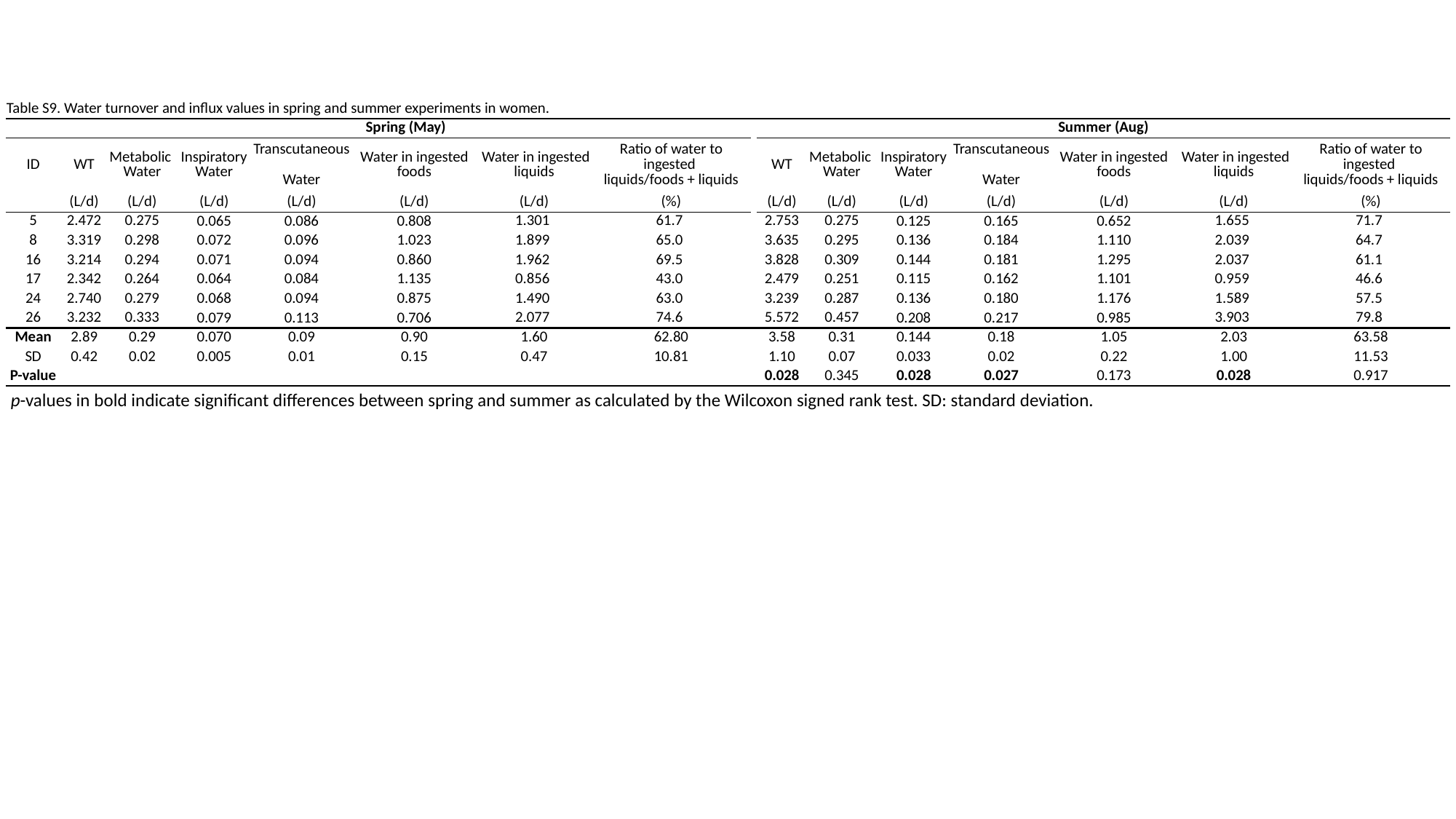

| Table S9. Water turnover and influx values in spring and summer experiments in women. | | | | | | | | | | | | | | | |
| --- | --- | --- | --- | --- | --- | --- | --- | --- | --- | --- | --- | --- | --- | --- | --- |
| | Spring (May) | | | | | | | | Summer (Aug) | | | | | | |
| ID | WT | Metabolic Water | Inspiratory Water | Transcutaneous Water | Water in ingested foods | Water in ingested liquids | Ratio of water to ingested liquids/foods + liquids | | WT | Metabolic Water | Inspiratory Water | Transcutaneous Water | Water in ingested foods | Water in ingested liquids | Ratio of water to ingested liquids/foods + liquids |
| | (L/d) | (L/d) | (L/d) | (L/d) | (L/d) | (L/d) | (%) | | (L/d) | (L/d) | (L/d) | (L/d) | (L/d) | (L/d) | (%) |
| 5 | 2.472 | 0.275 | 0.065 | 0.086 | 0.808 | 1.301 | 61.7 | | 2.753 | 0.275 | 0.125 | 0.165 | 0.652 | 1.655 | 71.7 |
| 8 | 3.319 | 0.298 | 0.072 | 0.096 | 1.023 | 1.899 | 65.0 | | 3.635 | 0.295 | 0.136 | 0.184 | 1.110 | 2.039 | 64.7 |
| 16 | 3.214 | 0.294 | 0.071 | 0.094 | 0.860 | 1.962 | 69.5 | | 3.828 | 0.309 | 0.144 | 0.181 | 1.295 | 2.037 | 61.1 |
| 17 | 2.342 | 0.264 | 0.064 | 0.084 | 1.135 | 0.856 | 43.0 | | 2.479 | 0.251 | 0.115 | 0.162 | 1.101 | 0.959 | 46.6 |
| 24 | 2.740 | 0.279 | 0.068 | 0.094 | 0.875 | 1.490 | 63.0 | | 3.239 | 0.287 | 0.136 | 0.180 | 1.176 | 1.589 | 57.5 |
| 26 | 3.232 | 0.333 | 0.079 | 0.113 | 0.706 | 2.077 | 74.6 | | 5.572 | 0.457 | 0.208 | 0.217 | 0.985 | 3.903 | 79.8 |
| Mean | 2.89 | 0.29 | 0.070 | 0.09 | 0.90 | 1.60 | 62.80 | | 3.58 | 0.31 | 0.144 | 0.18 | 1.05 | 2.03 | 63.58 |
| SD | 0.42 | 0.02 | 0.005 | 0.01 | 0.15 | 0.47 | 10.81 | | 1.10 | 0.07 | 0.033 | 0.02 | 0.22 | 1.00 | 11.53 |
| P-value | | | | | | | | | 0.028 | 0.345 | 0.028 | 0.027 | 0.173 | 0.028 | 0.917 |
p-values in bold indicate significant differences between spring and summer as calculated by the Wilcoxon signed rank test. SD: standard deviation.

## Slide 10
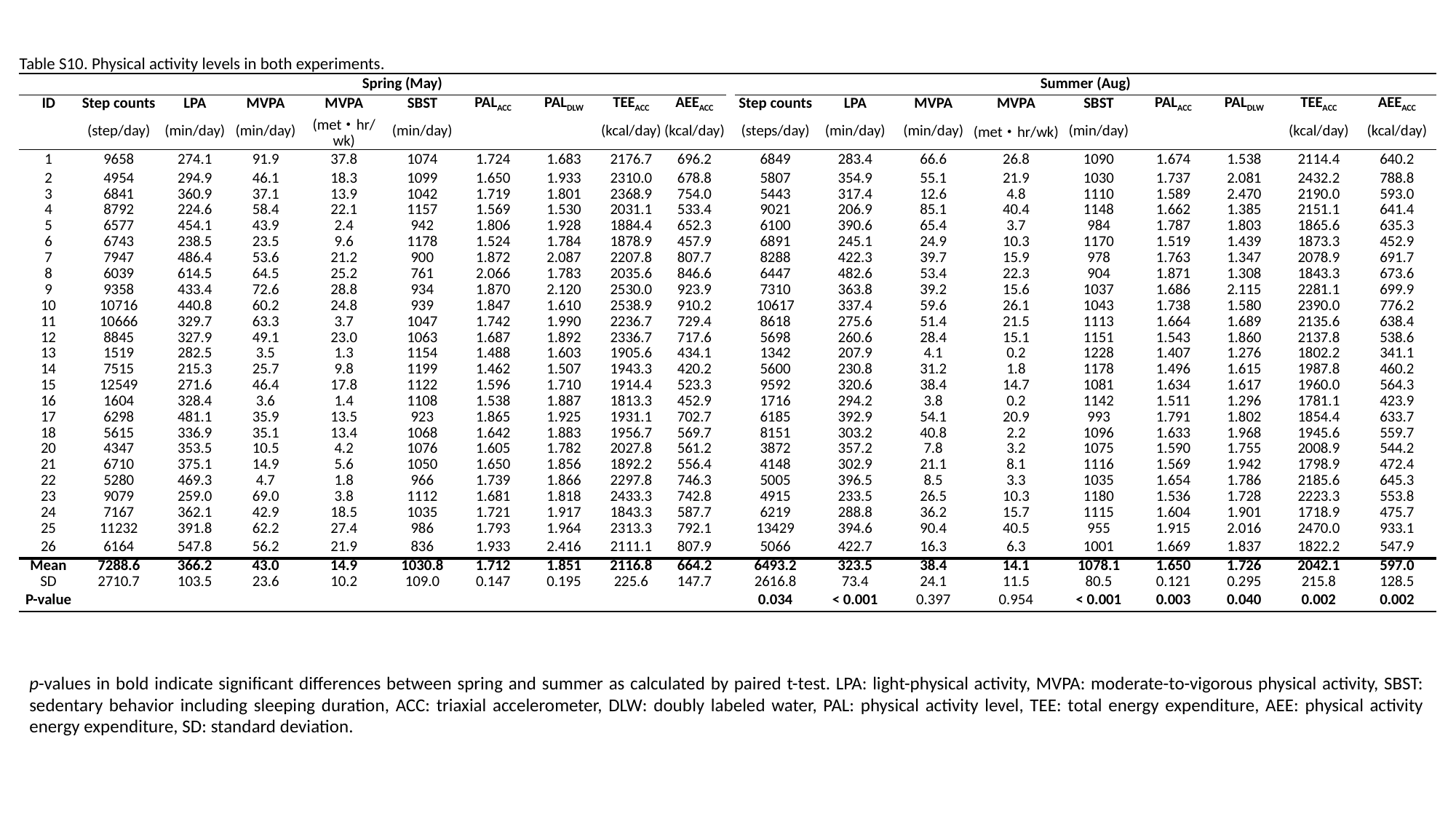

| Table S10. Physical activity levels in both experiments. | | | | | | | | | | | | | | | | | | | |
| --- | --- | --- | --- | --- | --- | --- | --- | --- | --- | --- | --- | --- | --- | --- | --- | --- | --- | --- | --- |
| | Spring (May) | | | | | | | | | | Summer (Aug) | | | | | | | | |
| ID | Step counts | LPA | MVPA | MVPA | SBST | PALACC | PALDLW | TEEACC | AEEACC | | Step counts | LPA | MVPA | MVPA | SBST | PALACC | PALDLW | TEEACC | AEEACC |
| | (step/day) | (min/day) | (min/day) | (met･hr/wk) | (min/day) | | | (kcal/day) | (kcal/day) | | (steps/day) | (min/day) | (min/day) | (met･hr/wk) | (min/day) | | | (kcal/day) | (kcal/day) |
| 1 | 9658 | 274.1 | 91.9 | 37.8 | 1074 | 1.724 | 1.683 | 2176.7 | 696.2 | | 6849 | 283.4 | 66.6 | 26.8 | 1090 | 1.674 | 1.538 | 2114.4 | 640.2 |
| 2 | 4954 | 294.9 | 46.1 | 18.3 | 1099 | 1.650 | 1.933 | 2310.0 | 678.8 | | 5807 | 354.9 | 55.1 | 21.9 | 1030 | 1.737 | 2.081 | 2432.2 | 788.8 |
| 3 | 6841 | 360.9 | 37.1 | 13.9 | 1042 | 1.719 | 1.801 | 2368.9 | 754.0 | | 5443 | 317.4 | 12.6 | 4.8 | 1110 | 1.589 | 2.470 | 2190.0 | 593.0 |
| 4 | 8792 | 224.6 | 58.4 | 22.1 | 1157 | 1.569 | 1.530 | 2031.1 | 533.4 | | 9021 | 206.9 | 85.1 | 40.4 | 1148 | 1.662 | 1.385 | 2151.1 | 641.4 |
| 5 | 6577 | 454.1 | 43.9 | 2.4 | 942 | 1.806 | 1.928 | 1884.4 | 652.3 | | 6100 | 390.6 | 65.4 | 3.7 | 984 | 1.787 | 1.803 | 1865.6 | 635.3 |
| 6 | 6743 | 238.5 | 23.5 | 9.6 | 1178 | 1.524 | 1.784 | 1878.9 | 457.9 | | 6891 | 245.1 | 24.9 | 10.3 | 1170 | 1.519 | 1.439 | 1873.3 | 452.9 |
| 7 | 7947 | 486.4 | 53.6 | 21.2 | 900 | 1.872 | 2.087 | 2207.8 | 807.7 | | 8288 | 422.3 | 39.7 | 15.9 | 978 | 1.763 | 1.347 | 2078.9 | 691.7 |
| 8 | 6039 | 614.5 | 64.5 | 25.2 | 761 | 2.066 | 1.783 | 2035.6 | 846.6 | | 6447 | 482.6 | 53.4 | 22.3 | 904 | 1.871 | 1.308 | 1843.3 | 673.6 |
| 9 | 9358 | 433.4 | 72.6 | 28.8 | 934 | 1.870 | 2.120 | 2530.0 | 923.9 | | 7310 | 363.8 | 39.2 | 15.6 | 1037 | 1.686 | 2.115 | 2281.1 | 699.9 |
| 10 | 10716 | 440.8 | 60.2 | 24.8 | 939 | 1.847 | 1.610 | 2538.9 | 910.2 | | 10617 | 337.4 | 59.6 | 26.1 | 1043 | 1.738 | 1.580 | 2390.0 | 776.2 |
| 11 | 10666 | 329.7 | 63.3 | 3.7 | 1047 | 1.742 | 1.990 | 2236.7 | 729.4 | | 8618 | 275.6 | 51.4 | 21.5 | 1113 | 1.664 | 1.689 | 2135.6 | 638.4 |
| 12 | 8845 | 327.9 | 49.1 | 23.0 | 1063 | 1.687 | 1.892 | 2336.7 | 717.6 | | 5698 | 260.6 | 28.4 | 15.1 | 1151 | 1.543 | 1.860 | 2137.8 | 538.6 |
| 13 | 1519 | 282.5 | 3.5 | 1.3 | 1154 | 1.488 | 1.603 | 1905.6 | 434.1 | | 1342 | 207.9 | 4.1 | 0.2 | 1228 | 1.407 | 1.276 | 1802.2 | 341.1 |
| 14 | 7515 | 215.3 | 25.7 | 9.8 | 1199 | 1.462 | 1.507 | 1943.3 | 420.2 | | 5600 | 230.8 | 31.2 | 1.8 | 1178 | 1.496 | 1.615 | 1987.8 | 460.2 |
| 15 | 12549 | 271.6 | 46.4 | 17.8 | 1122 | 1.596 | 1.710 | 1914.4 | 523.3 | | 9592 | 320.6 | 38.4 | 14.7 | 1081 | 1.634 | 1.617 | 1960.0 | 564.3 |
| 16 | 1604 | 328.4 | 3.6 | 1.4 | 1108 | 1.538 | 1.887 | 1813.3 | 452.9 | | 1716 | 294.2 | 3.8 | 0.2 | 1142 | 1.511 | 1.296 | 1781.1 | 423.9 |
| 17 | 6298 | 481.1 | 35.9 | 13.5 | 923 | 1.865 | 1.925 | 1931.1 | 702.7 | | 6185 | 392.9 | 54.1 | 20.9 | 993 | 1.791 | 1.802 | 1854.4 | 633.7 |
| 18 | 5615 | 336.9 | 35.1 | 13.4 | 1068 | 1.642 | 1.883 | 1956.7 | 569.7 | | 8151 | 303.2 | 40.8 | 2.2 | 1096 | 1.633 | 1.968 | 1945.6 | 559.7 |
| 20 | 4347 | 353.5 | 10.5 | 4.2 | 1076 | 1.605 | 1.782 | 2027.8 | 561.2 | | 3872 | 357.2 | 7.8 | 3.2 | 1075 | 1.590 | 1.755 | 2008.9 | 544.2 |
| 21 | 6710 | 375.1 | 14.9 | 5.6 | 1050 | 1.650 | 1.856 | 1892.2 | 556.4 | | 4148 | 302.9 | 21.1 | 8.1 | 1116 | 1.569 | 1.942 | 1798.9 | 472.4 |
| 22 | 5280 | 469.3 | 4.7 | 1.8 | 966 | 1.739 | 1.866 | 2297.8 | 746.3 | | 5005 | 396.5 | 8.5 | 3.3 | 1035 | 1.654 | 1.786 | 2185.6 | 645.3 |
| 23 | 9079 | 259.0 | 69.0 | 3.8 | 1112 | 1.681 | 1.818 | 2433.3 | 742.8 | | 4915 | 233.5 | 26.5 | 10.3 | 1180 | 1.536 | 1.728 | 2223.3 | 553.8 |
| 24 | 7167 | 362.1 | 42.9 | 18.5 | 1035 | 1.721 | 1.917 | 1843.3 | 587.7 | | 6219 | 288.8 | 36.2 | 15.7 | 1115 | 1.604 | 1.901 | 1718.9 | 475.7 |
| 25 | 11232 | 391.8 | 62.2 | 27.4 | 986 | 1.793 | 1.964 | 2313.3 | 792.1 | | 13429 | 394.6 | 90.4 | 40.5 | 955 | 1.915 | 2.016 | 2470.0 | 933.1 |
| 26 | 6164 | 547.8 | 56.2 | 21.9 | 836 | 1.933 | 2.416 | 2111.1 | 807.9 | | 5066 | 422.7 | 16.3 | 6.3 | 1001 | 1.669 | 1.837 | 1822.2 | 547.9 |
| Mean | 7288.6 | 366.2 | 43.0 | 14.9 | 1030.8 | 1.712 | 1.851 | 2116.8 | 664.2 | | 6493.2 | 323.5 | 38.4 | 14.1 | 1078.1 | 1.650 | 1.726 | 2042.1 | 597.0 |
| SD | 2710.7 | 103.5 | 23.6 | 10.2 | 109.0 | 0.147 | 0.195 | 225.6 | 147.7 | | 2616.8 | 73.4 | 24.1 | 11.5 | 80.5 | 0.121 | 0.295 | 215.8 | 128.5 |
| P-value | | | | | | | | | | | 0.034 | < 0.001 | 0.397 | 0.954 | < 0.001 | 0.003 | 0.040 | 0.002 | 0.002 |
p-values in bold indicate significant differences between spring and summer as calculated by paired t-test. LPA: light-physical activity, MVPA: moderate-to-vigorous physical activity, SBST: sedentary behavior including sleeping duration, ACC: triaxial accelerometer, DLW: doubly labeled water, PAL: physical activity level, TEE: total energy expenditure, AEE: physical activity energy expenditure, SD: standard deviation.

## Slide 11
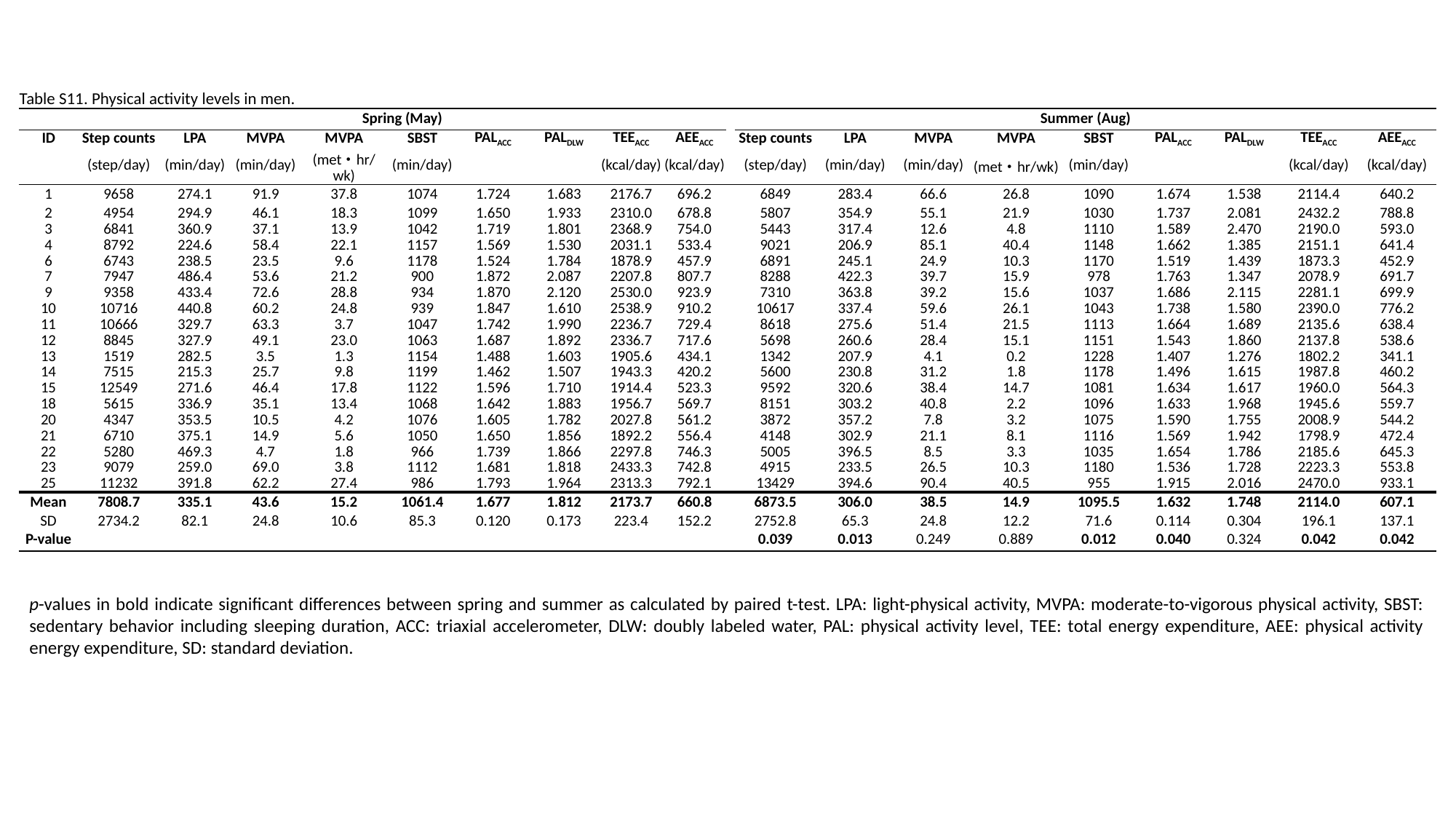

| Table S11. Physical activity levels in men. | | | | | | | | | | | | | | | | | | | |
| --- | --- | --- | --- | --- | --- | --- | --- | --- | --- | --- | --- | --- | --- | --- | --- | --- | --- | --- | --- |
| | Spring (May) | | | | | | | | | | Summer (Aug) | | | | | | | | |
| ID | Step counts | LPA | MVPA | MVPA | SBST | PALACC | PALDLW | TEEACC | AEEACC | | Step counts | LPA | MVPA | MVPA | SBST | PALACC | PALDLW | TEEACC | AEEACC |
| | (step/day) | (min/day) | (min/day) | (met･hr/wk) | (min/day) | | | (kcal/day) | (kcal/day) | | (step/day) | (min/day) | (min/day) | (met･hr/wk) | (min/day) | | | (kcal/day) | (kcal/day) |
| 1 | 9658 | 274.1 | 91.9 | 37.8 | 1074 | 1.724 | 1.683 | 2176.7 | 696.2 | | 6849 | 283.4 | 66.6 | 26.8 | 1090 | 1.674 | 1.538 | 2114.4 | 640.2 |
| 2 | 4954 | 294.9 | 46.1 | 18.3 | 1099 | 1.650 | 1.933 | 2310.0 | 678.8 | | 5807 | 354.9 | 55.1 | 21.9 | 1030 | 1.737 | 2.081 | 2432.2 | 788.8 |
| 3 | 6841 | 360.9 | 37.1 | 13.9 | 1042 | 1.719 | 1.801 | 2368.9 | 754.0 | | 5443 | 317.4 | 12.6 | 4.8 | 1110 | 1.589 | 2.470 | 2190.0 | 593.0 |
| 4 | 8792 | 224.6 | 58.4 | 22.1 | 1157 | 1.569 | 1.530 | 2031.1 | 533.4 | | 9021 | 206.9 | 85.1 | 40.4 | 1148 | 1.662 | 1.385 | 2151.1 | 641.4 |
| 6 | 6743 | 238.5 | 23.5 | 9.6 | 1178 | 1.524 | 1.784 | 1878.9 | 457.9 | | 6891 | 245.1 | 24.9 | 10.3 | 1170 | 1.519 | 1.439 | 1873.3 | 452.9 |
| 7 | 7947 | 486.4 | 53.6 | 21.2 | 900 | 1.872 | 2.087 | 2207.8 | 807.7 | | 8288 | 422.3 | 39.7 | 15.9 | 978 | 1.763 | 1.347 | 2078.9 | 691.7 |
| 9 | 9358 | 433.4 | 72.6 | 28.8 | 934 | 1.870 | 2.120 | 2530.0 | 923.9 | | 7310 | 363.8 | 39.2 | 15.6 | 1037 | 1.686 | 2.115 | 2281.1 | 699.9 |
| 10 | 10716 | 440.8 | 60.2 | 24.8 | 939 | 1.847 | 1.610 | 2538.9 | 910.2 | | 10617 | 337.4 | 59.6 | 26.1 | 1043 | 1.738 | 1.580 | 2390.0 | 776.2 |
| 11 | 10666 | 329.7 | 63.3 | 3.7 | 1047 | 1.742 | 1.990 | 2236.7 | 729.4 | | 8618 | 275.6 | 51.4 | 21.5 | 1113 | 1.664 | 1.689 | 2135.6 | 638.4 |
| 12 | 8845 | 327.9 | 49.1 | 23.0 | 1063 | 1.687 | 1.892 | 2336.7 | 717.6 | | 5698 | 260.6 | 28.4 | 15.1 | 1151 | 1.543 | 1.860 | 2137.8 | 538.6 |
| 13 | 1519 | 282.5 | 3.5 | 1.3 | 1154 | 1.488 | 1.603 | 1905.6 | 434.1 | | 1342 | 207.9 | 4.1 | 0.2 | 1228 | 1.407 | 1.276 | 1802.2 | 341.1 |
| 14 | 7515 | 215.3 | 25.7 | 9.8 | 1199 | 1.462 | 1.507 | 1943.3 | 420.2 | | 5600 | 230.8 | 31.2 | 1.8 | 1178 | 1.496 | 1.615 | 1987.8 | 460.2 |
| 15 | 12549 | 271.6 | 46.4 | 17.8 | 1122 | 1.596 | 1.710 | 1914.4 | 523.3 | | 9592 | 320.6 | 38.4 | 14.7 | 1081 | 1.634 | 1.617 | 1960.0 | 564.3 |
| 18 | 5615 | 336.9 | 35.1 | 13.4 | 1068 | 1.642 | 1.883 | 1956.7 | 569.7 | | 8151 | 303.2 | 40.8 | 2.2 | 1096 | 1.633 | 1.968 | 1945.6 | 559.7 |
| 20 | 4347 | 353.5 | 10.5 | 4.2 | 1076 | 1.605 | 1.782 | 2027.8 | 561.2 | | 3872 | 357.2 | 7.8 | 3.2 | 1075 | 1.590 | 1.755 | 2008.9 | 544.2 |
| 21 | 6710 | 375.1 | 14.9 | 5.6 | 1050 | 1.650 | 1.856 | 1892.2 | 556.4 | | 4148 | 302.9 | 21.1 | 8.1 | 1116 | 1.569 | 1.942 | 1798.9 | 472.4 |
| 22 | 5280 | 469.3 | 4.7 | 1.8 | 966 | 1.739 | 1.866 | 2297.8 | 746.3 | | 5005 | 396.5 | 8.5 | 3.3 | 1035 | 1.654 | 1.786 | 2185.6 | 645.3 |
| 23 | 9079 | 259.0 | 69.0 | 3.8 | 1112 | 1.681 | 1.818 | 2433.3 | 742.8 | | 4915 | 233.5 | 26.5 | 10.3 | 1180 | 1.536 | 1.728 | 2223.3 | 553.8 |
| 25 | 11232 | 391.8 | 62.2 | 27.4 | 986 | 1.793 | 1.964 | 2313.3 | 792.1 | | 13429 | 394.6 | 90.4 | 40.5 | 955 | 1.915 | 2.016 | 2470.0 | 933.1 |
| Mean | 7808.7 | 335.1 | 43.6 | 15.2 | 1061.4 | 1.677 | 1.812 | 2173.7 | 660.8 | | 6873.5 | 306.0 | 38.5 | 14.9 | 1095.5 | 1.632 | 1.748 | 2114.0 | 607.1 |
| SD | 2734.2 | 82.1 | 24.8 | 10.6 | 85.3 | 0.120 | 0.173 | 223.4 | 152.2 | | 2752.8 | 65.3 | 24.8 | 12.2 | 71.6 | 0.114 | 0.304 | 196.1 | 137.1 |
| P-value | | | | | | | | | | | 0.039 | 0.013 | 0.249 | 0.889 | 0.012 | 0.040 | 0.324 | 0.042 | 0.042 |
p-values in bold indicate significant differences between spring and summer as calculated by paired t-test. LPA: light-physical activity, MVPA: moderate-to-vigorous physical activity, SBST: sedentary behavior including sleeping duration, ACC: triaxial accelerometer, DLW: doubly labeled water, PAL: physical activity level, TEE: total energy expenditure, AEE: physical activity energy expenditure, SD: standard deviation.

## Slide 12
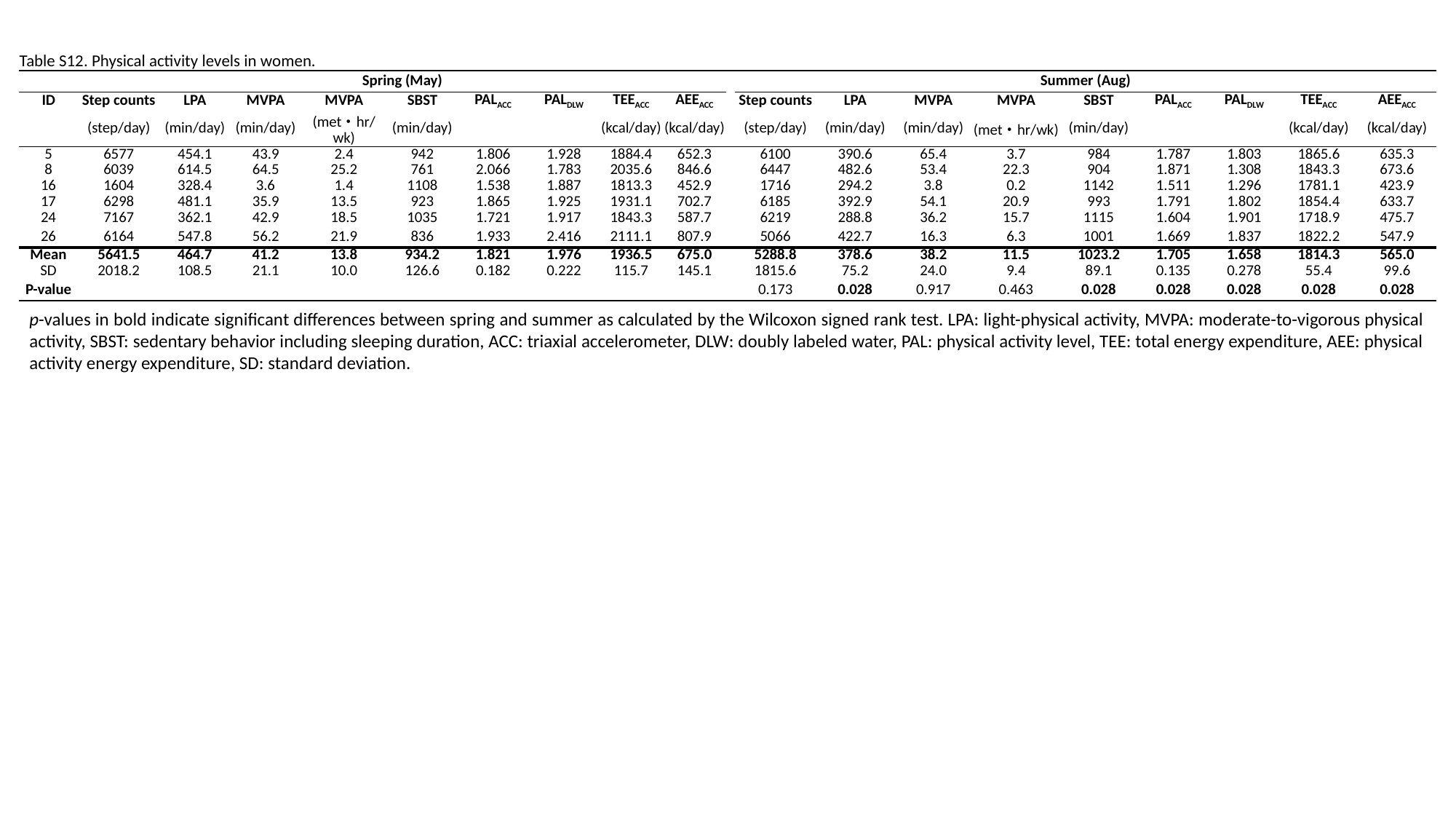

| Table S12. Physical activity levels in women. | | | | | | | | | | | | | | | | | | | |
| --- | --- | --- | --- | --- | --- | --- | --- | --- | --- | --- | --- | --- | --- | --- | --- | --- | --- | --- | --- |
| | Spring (May) | | | | | | | | | | Summer (Aug) | | | | | | | | |
| ID | Step counts | LPA | MVPA | MVPA | SBST | PALACC | PALDLW | TEEACC | AEEACC | | Step counts | LPA | MVPA | MVPA | SBST | PALACC | PALDLW | TEEACC | AEEACC |
| | (step/day) | (min/day) | (min/day) | (met･hr/wk) | (min/day) | | | (kcal/day) | (kcal/day) | | (step/day) | (min/day) | (min/day) | (met･hr/wk) | (min/day) | | | (kcal/day) | (kcal/day) |
| 5 | 6577 | 454.1 | 43.9 | 2.4 | 942 | 1.806 | 1.928 | 1884.4 | 652.3 | | 6100 | 390.6 | 65.4 | 3.7 | 984 | 1.787 | 1.803 | 1865.6 | 635.3 |
| 8 | 6039 | 614.5 | 64.5 | 25.2 | 761 | 2.066 | 1.783 | 2035.6 | 846.6 | | 6447 | 482.6 | 53.4 | 22.3 | 904 | 1.871 | 1.308 | 1843.3 | 673.6 |
| 16 | 1604 | 328.4 | 3.6 | 1.4 | 1108 | 1.538 | 1.887 | 1813.3 | 452.9 | | 1716 | 294.2 | 3.8 | 0.2 | 1142 | 1.511 | 1.296 | 1781.1 | 423.9 |
| 17 | 6298 | 481.1 | 35.9 | 13.5 | 923 | 1.865 | 1.925 | 1931.1 | 702.7 | | 6185 | 392.9 | 54.1 | 20.9 | 993 | 1.791 | 1.802 | 1854.4 | 633.7 |
| 24 | 7167 | 362.1 | 42.9 | 18.5 | 1035 | 1.721 | 1.917 | 1843.3 | 587.7 | | 6219 | 288.8 | 36.2 | 15.7 | 1115 | 1.604 | 1.901 | 1718.9 | 475.7 |
| 26 | 6164 | 547.8 | 56.2 | 21.9 | 836 | 1.933 | 2.416 | 2111.1 | 807.9 | | 5066 | 422.7 | 16.3 | 6.3 | 1001 | 1.669 | 1.837 | 1822.2 | 547.9 |
| Mean | 5641.5 | 464.7 | 41.2 | 13.8 | 934.2 | 1.821 | 1.976 | 1936.5 | 675.0 | | 5288.8 | 378.6 | 38.2 | 11.5 | 1023.2 | 1.705 | 1.658 | 1814.3 | 565.0 |
| SD | 2018.2 | 108.5 | 21.1 | 10.0 | 126.6 | 0.182 | 0.222 | 115.7 | 145.1 | | 1815.6 | 75.2 | 24.0 | 9.4 | 89.1 | 0.135 | 0.278 | 55.4 | 99.6 |
| P-value | | | | | | | | | | | 0.173 | 0.028 | 0.917 | 0.463 | 0.028 | 0.028 | 0.028 | 0.028 | 0.028 |
p-values in bold indicate significant differences between spring and summer as calculated by the Wilcoxon signed rank test. LPA: light-physical activity, MVPA: moderate-to-vigorous physical activity, SBST: sedentary behavior including sleeping duration, ACC: triaxial accelerometer, DLW: doubly labeled water, PAL: physical activity level, TEE: total energy expenditure, AEE: physical activity energy expenditure, SD: standard deviation.

## Slide 13
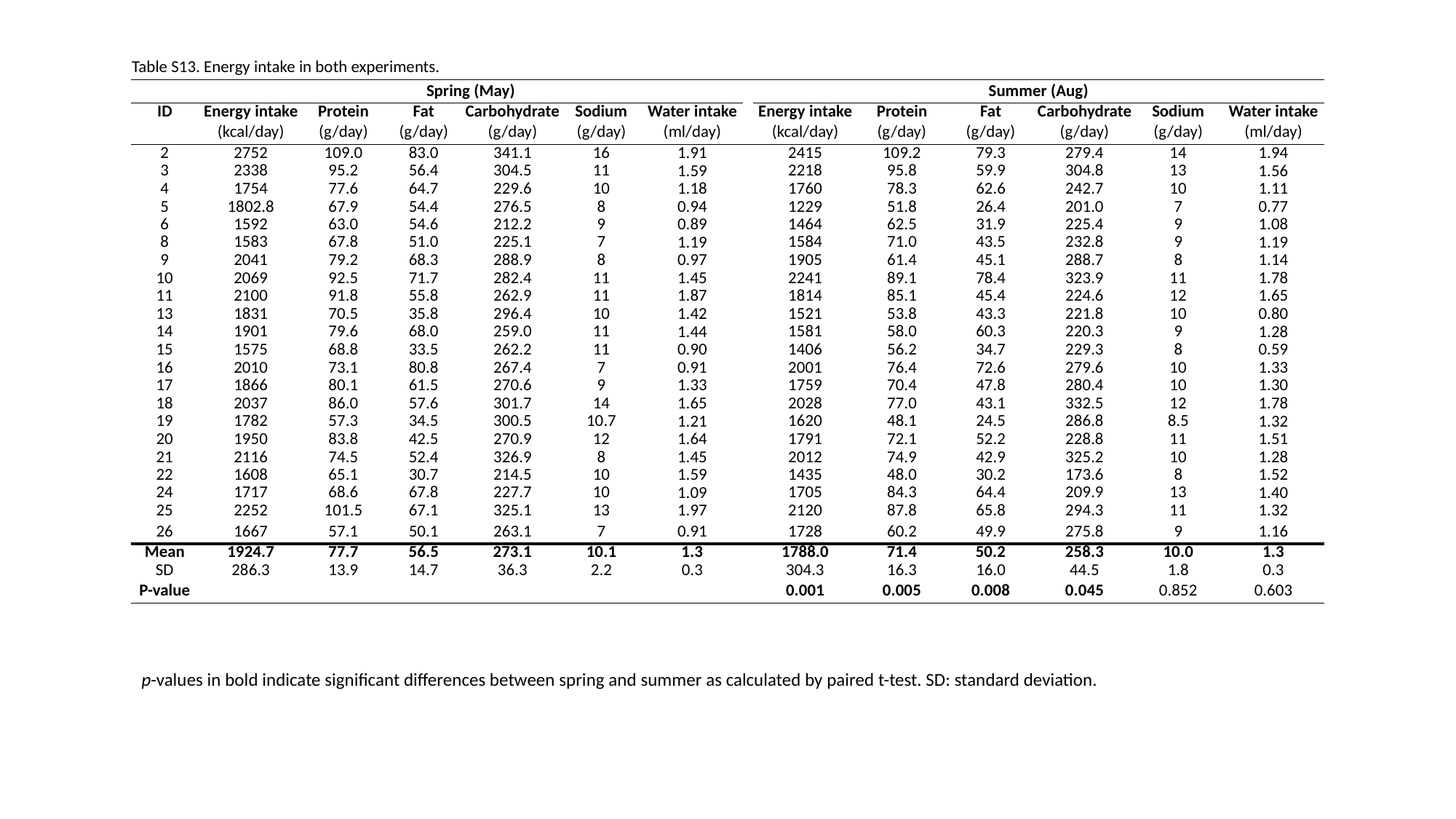

| Table S13. Energy intake in both experiments. | | | | | | | | | | | | | |
| --- | --- | --- | --- | --- | --- | --- | --- | --- | --- | --- | --- | --- | --- |
| | Spring (May) | | | | | | | Summer (Aug) | | | | | |
| ID | Energy intake | Protein | Fat | Carbohydrate | Sodium | Water intake | | Energy intake | Protein | Fat | Carbohydrate | Sodium | Water intake |
| | (kcal/day) | (g/day) | (g/day) | (g/day) | (g/day) | (ml/day) | | (kcal/day) | (g/day) | (g/day) | (g/day) | (g/day) | (ml/day) |
| 2 | 2752 | 109.0 | 83.0 | 341.1 | 16 | 1.91 | | 2415 | 109.2 | 79.3 | 279.4 | 14 | 1.94 |
| 3 | 2338 | 95.2 | 56.4 | 304.5 | 11 | 1.59 | | 2218 | 95.8 | 59.9 | 304.8 | 13 | 1.56 |
| 4 | 1754 | 77.6 | 64.7 | 229.6 | 10 | 1.18 | | 1760 | 78.3 | 62.6 | 242.7 | 10 | 1.11 |
| 5 | 1802.8 | 67.9 | 54.4 | 276.5 | 8 | 0.94 | | 1229 | 51.8 | 26.4 | 201.0 | 7 | 0.77 |
| 6 | 1592 | 63.0 | 54.6 | 212.2 | 9 | 0.89 | | 1464 | 62.5 | 31.9 | 225.4 | 9 | 1.08 |
| 8 | 1583 | 67.8 | 51.0 | 225.1 | 7 | 1.19 | | 1584 | 71.0 | 43.5 | 232.8 | 9 | 1.19 |
| 9 | 2041 | 79.2 | 68.3 | 288.9 | 8 | 0.97 | | 1905 | 61.4 | 45.1 | 288.7 | 8 | 1.14 |
| 10 | 2069 | 92.5 | 71.7 | 282.4 | 11 | 1.45 | | 2241 | 89.1 | 78.4 | 323.9 | 11 | 1.78 |
| 11 | 2100 | 91.8 | 55.8 | 262.9 | 11 | 1.87 | | 1814 | 85.1 | 45.4 | 224.6 | 12 | 1.65 |
| 13 | 1831 | 70.5 | 35.8 | 296.4 | 10 | 1.42 | | 1521 | 53.8 | 43.3 | 221.8 | 10 | 0.80 |
| 14 | 1901 | 79.6 | 68.0 | 259.0 | 11 | 1.44 | | 1581 | 58.0 | 60.3 | 220.3 | 9 | 1.28 |
| 15 | 1575 | 68.8 | 33.5 | 262.2 | 11 | 0.90 | | 1406 | 56.2 | 34.7 | 229.3 | 8 | 0.59 |
| 16 | 2010 | 73.1 | 80.8 | 267.4 | 7 | 0.91 | | 2001 | 76.4 | 72.6 | 279.6 | 10 | 1.33 |
| 17 | 1866 | 80.1 | 61.5 | 270.6 | 9 | 1.33 | | 1759 | 70.4 | 47.8 | 280.4 | 10 | 1.30 |
| 18 | 2037 | 86.0 | 57.6 | 301.7 | 14 | 1.65 | | 2028 | 77.0 | 43.1 | 332.5 | 12 | 1.78 |
| 19 | 1782 | 57.3 | 34.5 | 300.5 | 10.7 | 1.21 | | 1620 | 48.1 | 24.5 | 286.8 | 8.5 | 1.32 |
| 20 | 1950 | 83.8 | 42.5 | 270.9 | 12 | 1.64 | | 1791 | 72.1 | 52.2 | 228.8 | 11 | 1.51 |
| 21 | 2116 | 74.5 | 52.4 | 326.9 | 8 | 1.45 | | 2012 | 74.9 | 42.9 | 325.2 | 10 | 1.28 |
| 22 | 1608 | 65.1 | 30.7 | 214.5 | 10 | 1.59 | | 1435 | 48.0 | 30.2 | 173.6 | 8 | 1.52 |
| 24 | 1717 | 68.6 | 67.8 | 227.7 | 10 | 1.09 | | 1705 | 84.3 | 64.4 | 209.9 | 13 | 1.40 |
| 25 | 2252 | 101.5 | 67.1 | 325.1 | 13 | 1.97 | | 2120 | 87.8 | 65.8 | 294.3 | 11 | 1.32 |
| 26 | 1667 | 57.1 | 50.1 | 263.1 | 7 | 0.91 | | 1728 | 60.2 | 49.9 | 275.8 | 9 | 1.16 |
| Mean | 1924.7 | 77.7 | 56.5 | 273.1 | 10.1 | 1.3 | | 1788.0 | 71.4 | 50.2 | 258.3 | 10.0 | 1.3 |
| SD | 286.3 | 13.9 | 14.7 | 36.3 | 2.2 | 0.3 | | 304.3 | 16.3 | 16.0 | 44.5 | 1.8 | 0.3 |
| P-value | | | | | | | | 0.001 | 0.005 | 0.008 | 0.045 | 0.852 | 0.603 |
p-values in bold indicate significant differences between spring and summer as calculated by paired t-test. SD: standard deviation.

## Slide 14
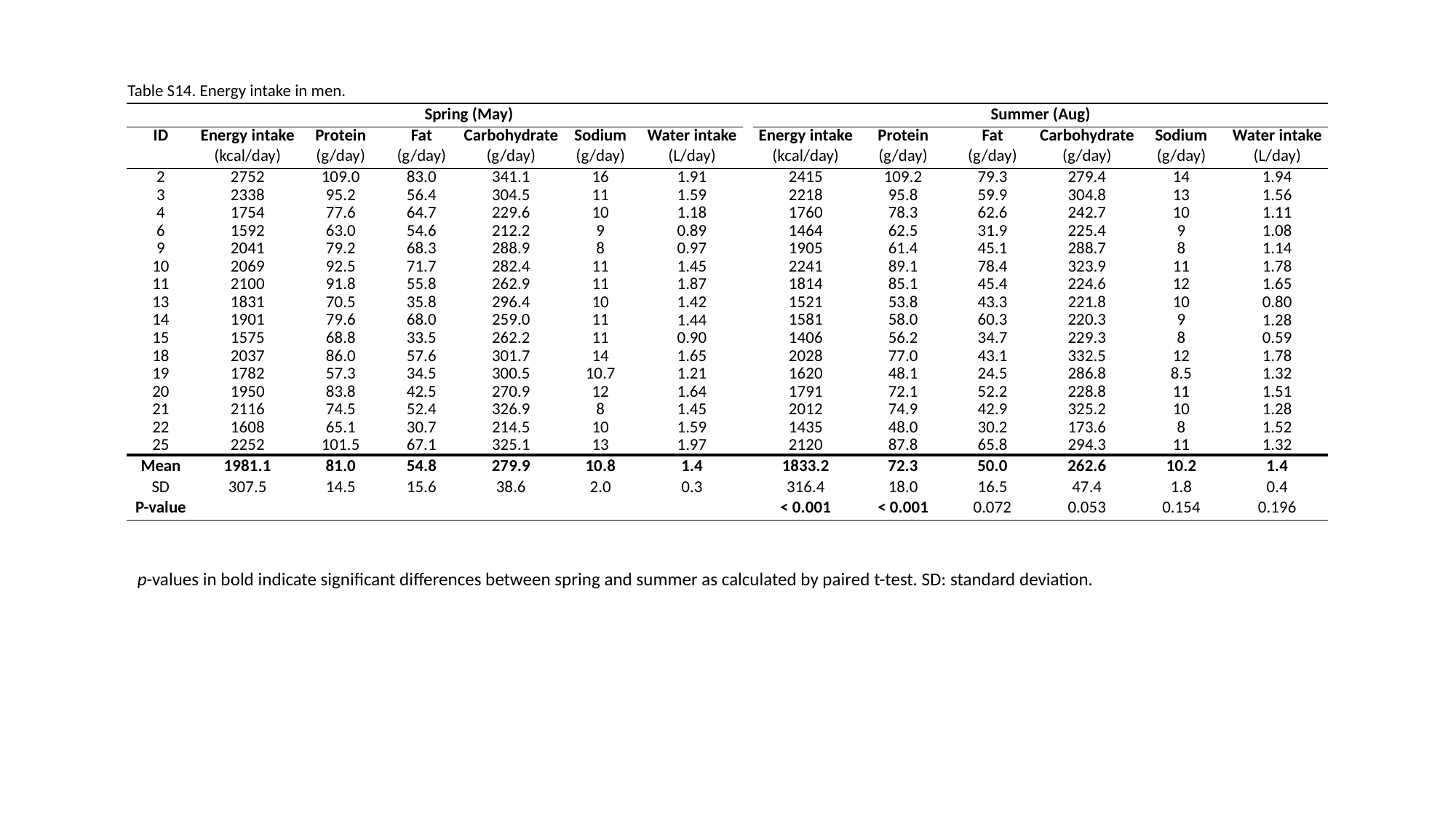

| Table S14. Energy intake in men. | | | | | | | | | | | | | |
| --- | --- | --- | --- | --- | --- | --- | --- | --- | --- | --- | --- | --- | --- |
| | Spring (May) | | | | | | | Summer (Aug) | | | | | |
| ID | Energy intake | Protein | Fat | Carbohydrate | Sodium | Water intake | | Energy intake | Protein | Fat | Carbohydrate | Sodium | Water intake |
| | (kcal/day) | (g/day) | (g/day) | (g/day) | (g/day) | (L/day) | | (kcal/day) | (g/day) | (g/day) | (g/day) | (g/day) | (L/day) |
| 2 | 2752 | 109.0 | 83.0 | 341.1 | 16 | 1.91 | | 2415 | 109.2 | 79.3 | 279.4 | 14 | 1.94 |
| 3 | 2338 | 95.2 | 56.4 | 304.5 | 11 | 1.59 | | 2218 | 95.8 | 59.9 | 304.8 | 13 | 1.56 |
| 4 | 1754 | 77.6 | 64.7 | 229.6 | 10 | 1.18 | | 1760 | 78.3 | 62.6 | 242.7 | 10 | 1.11 |
| 6 | 1592 | 63.0 | 54.6 | 212.2 | 9 | 0.89 | | 1464 | 62.5 | 31.9 | 225.4 | 9 | 1.08 |
| 9 | 2041 | 79.2 | 68.3 | 288.9 | 8 | 0.97 | | 1905 | 61.4 | 45.1 | 288.7 | 8 | 1.14 |
| 10 | 2069 | 92.5 | 71.7 | 282.4 | 11 | 1.45 | | 2241 | 89.1 | 78.4 | 323.9 | 11 | 1.78 |
| 11 | 2100 | 91.8 | 55.8 | 262.9 | 11 | 1.87 | | 1814 | 85.1 | 45.4 | 224.6 | 12 | 1.65 |
| 13 | 1831 | 70.5 | 35.8 | 296.4 | 10 | 1.42 | | 1521 | 53.8 | 43.3 | 221.8 | 10 | 0.80 |
| 14 | 1901 | 79.6 | 68.0 | 259.0 | 11 | 1.44 | | 1581 | 58.0 | 60.3 | 220.3 | 9 | 1.28 |
| 15 | 1575 | 68.8 | 33.5 | 262.2 | 11 | 0.90 | | 1406 | 56.2 | 34.7 | 229.3 | 8 | 0.59 |
| 18 | 2037 | 86.0 | 57.6 | 301.7 | 14 | 1.65 | | 2028 | 77.0 | 43.1 | 332.5 | 12 | 1.78 |
| 19 | 1782 | 57.3 | 34.5 | 300.5 | 10.7 | 1.21 | | 1620 | 48.1 | 24.5 | 286.8 | 8.5 | 1.32 |
| 20 | 1950 | 83.8 | 42.5 | 270.9 | 12 | 1.64 | | 1791 | 72.1 | 52.2 | 228.8 | 11 | 1.51 |
| 21 | 2116 | 74.5 | 52.4 | 326.9 | 8 | 1.45 | | 2012 | 74.9 | 42.9 | 325.2 | 10 | 1.28 |
| 22 | 1608 | 65.1 | 30.7 | 214.5 | 10 | 1.59 | | 1435 | 48.0 | 30.2 | 173.6 | 8 | 1.52 |
| 25 | 2252 | 101.5 | 67.1 | 325.1 | 13 | 1.97 | | 2120 | 87.8 | 65.8 | 294.3 | 11 | 1.32 |
| Mean | 1981.1 | 81.0 | 54.8 | 279.9 | 10.8 | 1.4 | | 1833.2 | 72.3 | 50.0 | 262.6 | 10.2 | 1.4 |
| SD | 307.5 | 14.5 | 15.6 | 38.6 | 2.0 | 0.3 | | 316.4 | 18.0 | 16.5 | 47.4 | 1.8 | 0.4 |
| P-value | | | | | | | | < 0.001 | < 0.001 | 0.072 | 0.053 | 0.154 | 0.196 |
p-values in bold indicate significant differences between spring and summer as calculated by paired t-test. SD: standard deviation.

## Slide 15
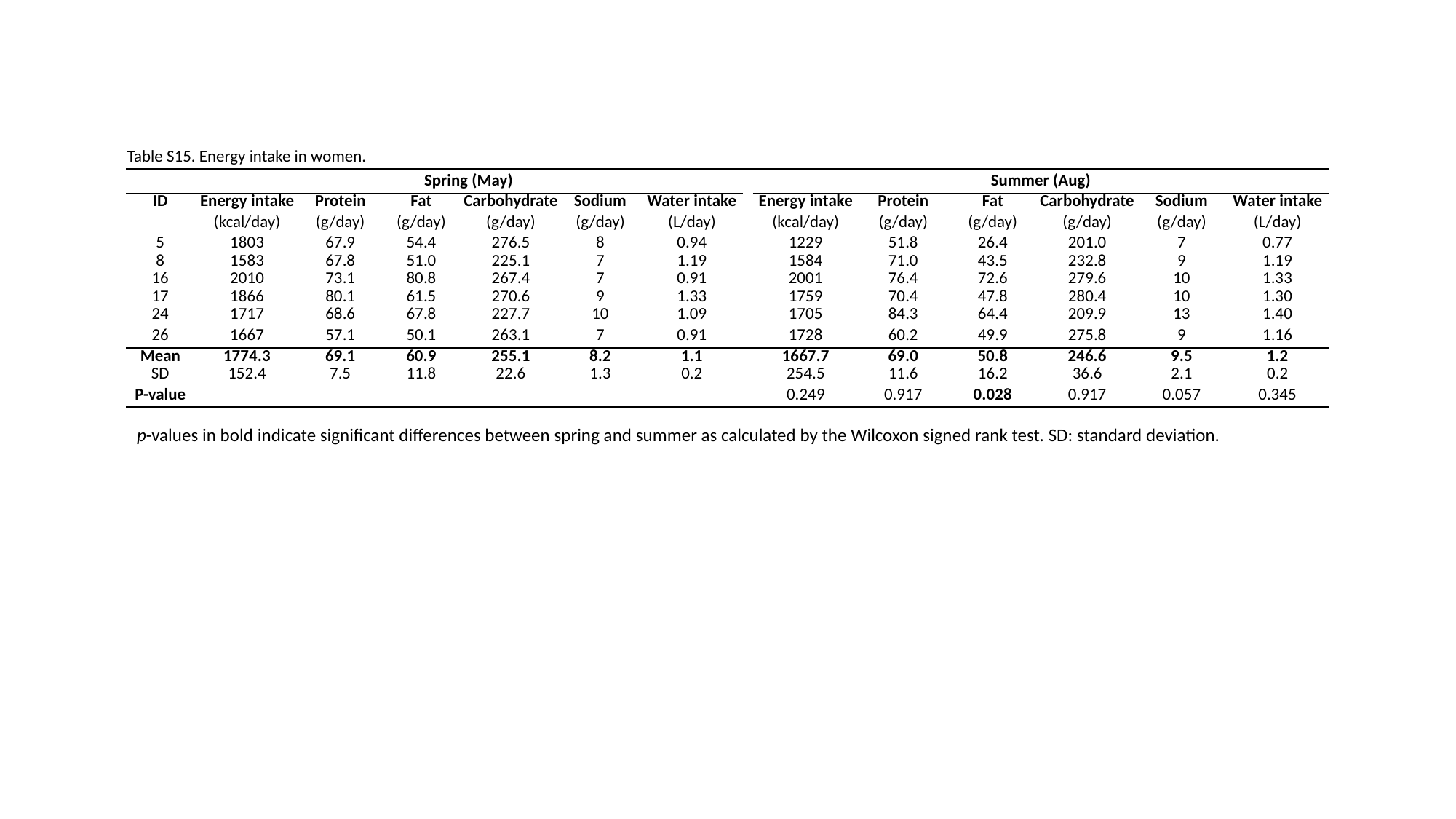

| Table S15. Energy intake in women. | | | | | | | | | | | | | |
| --- | --- | --- | --- | --- | --- | --- | --- | --- | --- | --- | --- | --- | --- |
| | Spring (May) | | | | | | | Summer (Aug) | | | | | |
| ID | Energy intake | Protein | Fat | Carbohydrate | Sodium | Water intake | | Energy intake | Protein | Fat | Carbohydrate | Sodium | Water intake |
| | (kcal/day) | (g/day) | (g/day) | (g/day) | (g/day) | (L/day) | | (kcal/day) | (g/day) | (g/day) | (g/day) | (g/day) | (L/day) |
| 5 | 1803 | 67.9 | 54.4 | 276.5 | 8 | 0.94 | | 1229 | 51.8 | 26.4 | 201.0 | 7 | 0.77 |
| 8 | 1583 | 67.8 | 51.0 | 225.1 | 7 | 1.19 | | 1584 | 71.0 | 43.5 | 232.8 | 9 | 1.19 |
| 16 | 2010 | 73.1 | 80.8 | 267.4 | 7 | 0.91 | | 2001 | 76.4 | 72.6 | 279.6 | 10 | 1.33 |
| 17 | 1866 | 80.1 | 61.5 | 270.6 | 9 | 1.33 | | 1759 | 70.4 | 47.8 | 280.4 | 10 | 1.30 |
| 24 | 1717 | 68.6 | 67.8 | 227.7 | 10 | 1.09 | | 1705 | 84.3 | 64.4 | 209.9 | 13 | 1.40 |
| 26 | 1667 | 57.1 | 50.1 | 263.1 | 7 | 0.91 | | 1728 | 60.2 | 49.9 | 275.8 | 9 | 1.16 |
| Mean | 1774.3 | 69.1 | 60.9 | 255.1 | 8.2 | 1.1 | | 1667.7 | 69.0 | 50.8 | 246.6 | 9.5 | 1.2 |
| SD | 152.4 | 7.5 | 11.8 | 22.6 | 1.3 | 0.2 | | 254.5 | 11.6 | 16.2 | 36.6 | 2.1 | 0.2 |
| P-value | | | | | | | | 0.249 | 0.917 | 0.028 | 0.917 | 0.057 | 0.345 |
p-values in bold indicate significant differences between spring and summer as calculated by the Wilcoxon signed rank test. SD: standard deviation.
